# Machine learning meets psoriasis: identifying key lactylation biomarkers as potential targets for diagnosis and therapies

**DOI:** 10.3389/fimmu.2026.1791693

**Published:** 2026-03-13

**Authors:** Shangkun Li, Yan Qi, Dan Qiao, Xuping Hu, Liping Yao, Xueling Cui

**Affiliations:** 1 Department of Cell Biology and Medical Genetics, College of Basic Medical Sciences, Jilin University, Changchun, Jilin, China; 2 Department of Immunology, College of Basic Medical Sciences, Jilin University, Changchun, Jilin, China; 3 Department of Physiology, College of Basic Medical Sciences, Jilin University, Changchun, Jilin, China

**Keywords:** ENO1, lactylation-related biomarkers, machine learning, MPHOSPH6, psoriasis

## Abstract

**Background:**

Psoriasis is a long-term autoimmune skin condition marked by repeated inflammation. Recent findings indicate that affected skin in psoriasis shows increased aerobic glycolysis and lactate buildup, suggesting that protein lactylation may play a role in the disease. However, biomarkers related to lactylation for diagnosing and treating psoriasis remain poorly defined.

**Methods:**

Initially, genes with altered expression in psoriasis were identified. Key gene modules from Weighted Gene Co-expression Network Analysis (WGCNA) were used to pinpoint psoriasis-associated genes. These genes were then intersected with lactylation-related genes. Random Forest and LASSO regression algorithms selected lactylation-related biomarkers. Mouse psoriasis models were created using imiquimod to validate key gene expression. The immune microenvironment in psoriasis lesions was analyzed with CIBERSORT. Regulatory networks of miRNAs(microRNAs)-genes and TFs(Transcription Factors)-genes were built using NetworkAnalyst. Potential drugs targeting these biomarkers were predicted via the DSigDB database, and their expression and distribution were visualized in single-cell sequencing data. Finally, two-sample Mendelian randomization and summary data-based Mendelian randomization were performed to investigate the causal relationship between the biomarkers and psoriasis.

**Results:**

A total of 1,623 key genes associated with psoriasis were identified through differential gene screening and WGCNA analysis. Among these, 26 were related to lactylation. Machine learning pinpointed MPHOSPH6, ENO1, MKI67, and FABP5 as lactylation-related biomarkers for psoriasis, with ROC curves confirming their strong diagnostic capabilities. RT-qPCR experiments validated their reliability, and immune infiltration analysis showed significant correlations with immune cells. Additionally, 103 drugs targeting these biomarkers were found in the DSigDB database. Mendelian randomization analysis suggested that high levels of MPHOSPH6 and ENO1 are risk factors for psoriasis.

**Conclusion:**

MPHOSPH6, ENO1, MKI67, and FABP5 are identified as lactylation-related biomarkers for psoriasis, with MPHOSPH6 and ENO1 overexpression posing as risk factors. These findings offer potential new diagnostic and therapeutic targets for the disease.

## 1 Introduction

Psoriasis is a chronic, immune-mediated inflammatory skin disease affecting approximately 2–3% of the global population (1). It is characterized by keratinocyte hyperproliferation, aberrant differentiation, and sustained activation of the IL-23/IL-17 immune axis (2, 3). Although biologics targeting TNF-α, IL-17, and IL-23 have substantially improved clinical outcomes, a considerable proportion of patients exhibit incomplete responses, relapse after treatment discontinuation, or gradual loss of efficacy over time (4, 5). These clinical challenges suggest that additional regulatory layers beyond canonical cytokine signaling contribute to disease persistence and heterogeneity.

Recent advances in immunometabolism have revealed that psoriatic lesions exhibit profound metabolic reprogramming resembling a “Warburg-like” phenotype (6). Keratinocytes demonstrate enhanced glycolysis, increased lactate production, and activation of hypoxia-inducible factor-1α (HIF-1α) signaling (7, 8). Elevated lactate levels in psoriatic skin are no longer regarded as mere metabolic byproducts; instead, lactate functions as a bioactive metabolite capable of shaping immune responses and epithelial behavior (9). Accumulating evidence indicates that lactate contributes to sustaining type 17 inflammation and reinforcing IL-17-driven transcriptional programs, thereby promoting chronic disease progression (10).

A major conceptual breakthrough in this field was the identification of lysine lactylation (Kla), a novel lactate-derived post-translational modification that directly links cellular metabolic state to epigenetic regulation (11). Histone lactylation has been shown to modulate gene expression programs in immune cells, influencing macrophage polarization and inflammatory responses (11, 12). Emerging data suggest that similar mechanisms operate in psoriasis, where metabolic rewiring and immune activation are tightly interconnected.

Recent mechanistic studies have begun to define specific lactylation events in psoriatic pathology. Notably, IL-17A signaling has been reported to promote histone lactylation in keratinocytes via a KLK8–IL-17 receptor–HAT1 regulatory axis, establishing a positive feedback loop that amplifies inflammatory gene transcription and keratinocyte proliferation (13). Increased lactate availability has also been associated with reduced responsiveness to anti-IL-17A therapy, whereas pharmacological inhibition of lactate transport enhanced therapeutic efficacy in experimental models (13, 14). These findings position lactylation as a potential mediator of both disease exacerbation and therapeutic resistance.

Beyond keratinocytes, lactate acts as a metabolic communicator between epidermal cells and infiltrating immune populations. Epidermis-derived lactate can be transported via monocarboxylate transporters (MCTs) into macrophages and other immune cells, reshaping their metabolic programs and inflammatory phenotypes (14, 15). Thus, psoriasis may represent a metabolically coordinated inflammatory ecosystem in which lactate-driven epigenetic remodeling sustains immune–epithelial cross-talk.

Despite these advances, the comprehensive lactylation landscape in psoriasis remains incompletely characterized. The identity of key lactylated proteins, the relative contributions of histone versus non-histone lactylation, and inter-patient heterogeneity in lactylation signatures are still poorly understood (16). Traditional experimental approaches, while indispensable, may not fully capture the multidimensional complexity of immune–metabolic regulatory networks.

In this context, machine learning (ML) offers a transformative opportunity to accelerate biomarker discovery and mechanistic inference. By integrating transcriptomic, epigenomic, proteomic, and clinical datasets, ML algorithms can identify latent patterns, prioritize candidate lactylation-related regulators, and uncover nonlinear interactions within inflammatory circuits (17, 18). In this study, we used bioinformatics and machine learning to identify potential biomarkers linked to lactylation in psoriasis. We analyzed psoriasis-related datasets from the GEO database, identifying key genes through differential expression analysis and Weighted Gene Co-expression Network Analysis (WGCNA). These genes were then compared with lactylation-related genes (LRGs) from previous studies to find lactylation-related key genes, whose proteins can undergo lactylation. Using two machine learning algorithms, we identified crucial diagnostic biomarkers and developed a nomogram model to improve predictive accuracy and clinical relevance. Additionally, we performed two-sample Mendelian randomization (TSMR) and summary-data-based Mendelian randomization (SMR) analyses to explore the causal relationship between these biomarkers and psoriasis. Our research identifies lactylation-related biomarkers in psoriasis and explores their causal links to the disease, offering new therapeutic and diagnostic targets and encouraging further study of lactylated proteins in psoriasis pathogenesis.

## 2 Materials and methods

### 2.1 Data collection

The analysis used three independent psoriasis gene expression datasets from the Gen Expression Omnibus (GEO) repository: GSE13355, GSE14905, and GSE121212. Only healthy control and lesional skin samples were included, excluding non-lesional samples. GSE13355 (64 normal, 58 psoriatic samples) and GSE14905 (21 normal, 28 psoriatic samples) were microarray-based on the GPL570 platform, while GSE121212 (38 normal, 28 psoriatic samples) was RNA-seq-based on the GPL16791 platform. GSE13355 was the training set, with GSE14905 and GSE121212 as validation sets. Single-cell RNA-seq data from GSE151177 (GPL18573) included five normal and five lesional samples. Lactylation-related genes, totaling 327, were sourced from previous literature (19).

### 2.2 Screening of differentially expressed genes (DEGs) and enrichment analysis

In the GSE13355 dataset, the “limma” R package was used to identify significantly altered gene expressions in psoriasis, applying thresholds of |logFC| > 0.5 and *p*-value < 0.05. A volcano plot was created with “ggplot2”, and genes with |logFC| > 3 and p-value < 0.005 were labeled. A hierarchical clustering heatmap of the top 50 significant genes was generated using “pheatmap”. Functional annotation of DEGs was conducted through GO and KEGG enrichment analyses with “clusterProfiler”, and results were visualized using “enrichplot”.

### 2.3 Weighted gene co-expression network analysis

To identify key gene modules involved in psoriasis pathogenesis, WGCNA was conducted using the WGCNA package in R. Outlier samples were removed with the “cutreeStatic” function, setting “cutHeight” at 76 and “minSize” at 10. The “pickSoftThreshold” function determined the soft threshold for network construction. Using this threshold, the “blockwiseModules” function identified modules with “minModuleSize” at 60 and “mergeCutHeight” at 0.25. The hierarchical clustering dendrogram was visualized with “plotDendroAndColors”. Pearson’s correlation analysis assessed the relationship between gene modules and clinical traits, displayed via heatmap. “Gene Significance” (GS) evaluated gene-trait associations, while “Module Membership” (MM) described connectivity patterns.

### 2.4 Selection of key diagnostic genes using machine learning and the construction of a nomogram model

We implemented two machine learning algorithms to identify consensus genes through intersection analysis. Using the “randomForest” R package, we conducted random forest analysis to handle high-dimensional data, employing Gini index reduction to determine variable importance and selecting the top 5 genes for further analysis. LASSO regression was performed with the “glmnet” package, using L1-penalized logistic regression and selecting the regularization parameter lambda via 10-fold cross-validation. This method automatically selects features by shrinking non-informative variables to zero. Genes selected by both methods were identified as consensus key genes. We then used the “rms” R package to construct a clinical nomogram based on these key diagnostic genes.

### 2.5 Generating ROC curves and box plots for visualization

The diagnostic performance of candidate genes was evaluated using ROC curve analysis via the “pROC” package in R, with AUC values indicating each gene’s ability to classify diseases. Gene expression patterns were visualized with boxplots from the “ggpubr” package, allowing comparison of transcriptional profiles across sample groups.

### 2.6 Construction of mouse psoriasis models

Male BALB/c mice were obtained from Changchun Yisi Experimental Animal Technology Co., Ltd. and kept in a controlled environment (22 ± 2 °C, 45-55% humidity) with a 12-hour light/dark cycle. They had free access to food and water. Before starting the treatment, a 2.5×3 cm area of dorsal skin was shaved aseptically. The experiment involved two groups (n=5 each). A psoriatic model was induced by applying 62.5 mg of 5% imiquimod cream daily for seven days, while the control group was untreated. Disease progression was photographed daily, and lesion severity was assessed using a modified Psoriasis Area and Severity Index (PASI) based on erythema, scaling, and skin thickening, each rated from 0 (none) to 4 (very severe). The total of these scores formed the PASI score (20).

### 2.7 HE staining

Paraffin-embedded tissue sections were dewaxed, rehydrated, and stained with hematoxylin for 1 minute, then rinsed. They were treated with 1% hydrochloric acid alcohol for 20 seconds, rinsed, and treated with 1% ammonia water for 30 seconds, followed by another rinse. The sections were dehydrated in 85% and 95% ethanol for 5 minutes each, stained with eosin for 30 seconds, and rinsed with absolute ethanol. Dehydration was repeated with absolute ethanol for 5 minutes, followed by xylene treatment and mounting with neutral balsam. The sections were then examined for pathological changes under an optical microscope.

### 2.8 Reverse transcription–quantitative PCR

RNA was extracted from tissue using TRIzol, and cDNA was synthesized via reverse transcription with Prime-Script RTase. Real-time PCR was then performed using the cDNA and specific primers (details in **Supplementary Table 1**). The 2^−ΔΔCt^ method measured relative mRNA expression, with GAPDH as the reference gene.

### 2.9 Immune infiltration

We used the CIBERSORT method to quantify immune cells in tissue samples, focusing only on statistically significant specimens (P-value < 0.05). Violin plots illustrated differences in immune cell infiltration between diseased and healthy groups. We also explored correlations between key diagnostic biomarkers and immune cells using Pearson’s analysis, with results shown in a heatmap.

### 2.10 Construction of miRNAs-genes and TFs-genes regulatory networks, and prediction of drugs in the DSigDB database

Regulatory networks of miRNAs-genes and TFs-genes were created via NetworkAnalyst. Gene-targeting microRNAs came from TarBase, and transcription factors from JASPAR. Drug predictions for key biomarkers were made using DSigDB on Enrichr (https://maayanlab.cloud/Enrichr/).

### 2.11 Single-cell sequencing data analysis

Single-cell sequencing data was processed using the “Seurat” R package. Quality control retained cells with: (1) 200–6000 genes, (2) >500 detected counts, (3) ≤10% mitochondrial genes, and (4) ≤1% hemoglobin genes. Gene expression data was normalized with “NormalizeData”, and the “vst” method identified the top 2,000 variable genes. Data scaling was done using “ScaleData” before dimensionality reduction with “RunPCA”. Batch effects were removed using “RunHarmony”. Cell clustering was performed with “FindNeighbors” and “FindClusters”. Initial cell annotation used the “singleR” R package, followed by manual corrections using the CellMarker database for accuracy.

### 2.12 Two-sample mendelian randomization

We obtained the psoriasis GWAS dataset (GWAS ID: ukb-b-10537) from the IEU OpenGWAS repository, including 5,314 cases and 457,619 controls. eQTL data for relevant biomarkers came from the eQTLGen database. Using the TwoSampleMR R package, we performed two-sample Mendelian randomization (MR) analyses. We selected SNPs with strong gene associations (p < 5.0×10^−8^) and removed those in linkage disequilibrium (R² < 0.001, 10,000 kb window). SNPs with F-statistics below 10 were excluded to avoid weak instrument bias. Our main analysis used inverse variance weighted (IVW) estimation to explore causal links between gene expression and psoriasis risk, with further validation through MR Egger, weighted median, simple mode, and weighted mode analyses. Sensitivity analyses included Cochrane’s Q test for heterogeneity and MR-Egger intercept testing for horizontal pleiotropy.

### 2.13 Summary-data-based mendelian randomization

The SMR software was used to perform an analysis that employs top cis-eQTL SNVs as instrumental variables, combining eQTL and GWAS data to investigate causal links between gene expression and disease. The HEIDI test helped differentiate causal associations from linkage disequilibrium. A causal relationship between gene expression and psoriasis was suggested by results with *p_SMR_* < 0.05 and *p_HEIDI_* > 0.05.

### 2.14 Statistical analysis

Statistical analyses were performed using R (version 4.4.3). Student’s t-test assessed differences in normally distributed continuous data, while the Wilcoxon rank-sum test was used for non-normal data. Two-tailed p-values were reported, with significance at *p* < 0.05. Pearson’s correlation coefficient evaluated variable associations.

## 3 Results

### 3.1 Identification and functional analysis of DEGs in psoriasis

**Figure 1:**
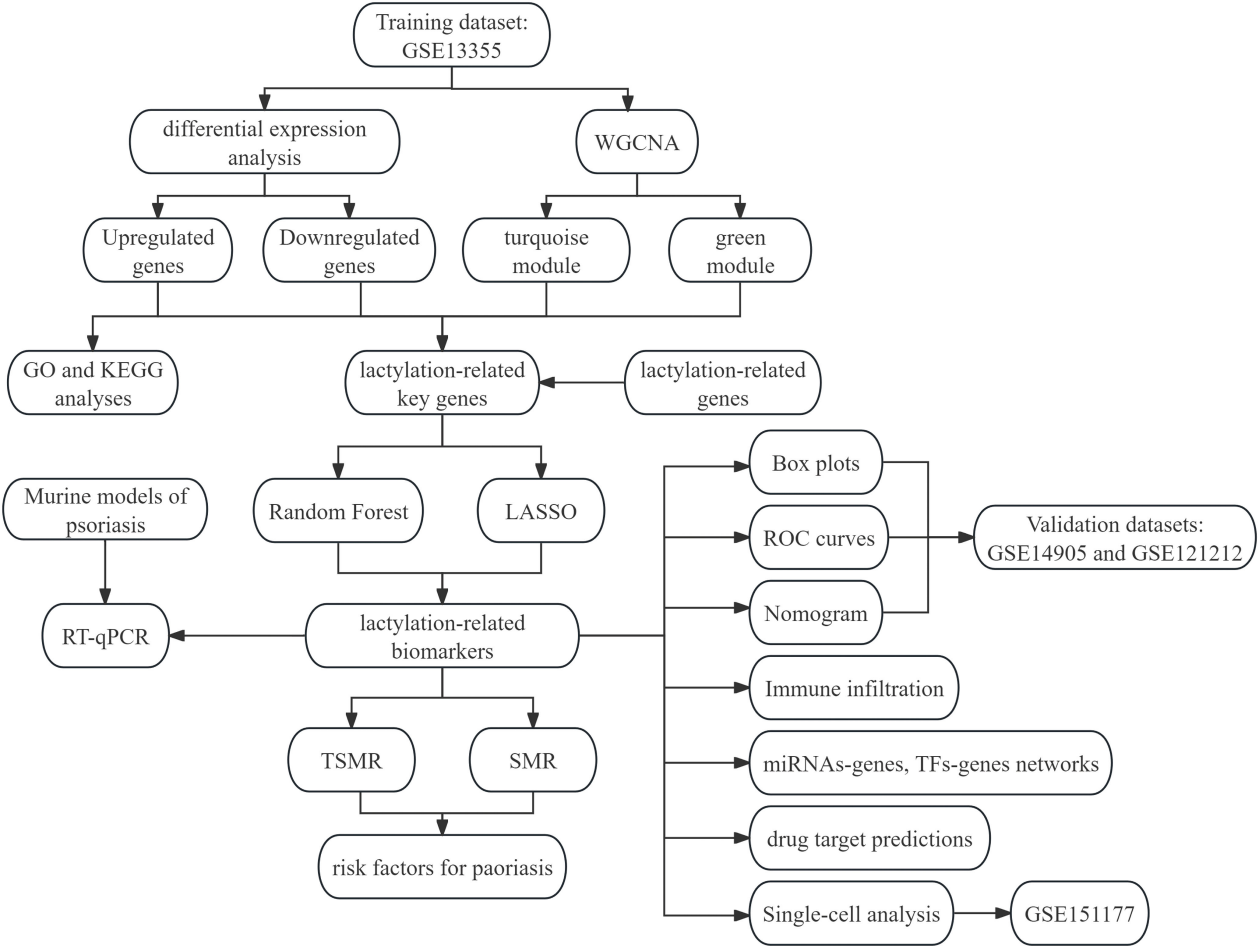
Flowchart of bioinformatic analyses.

**Figure 2:**
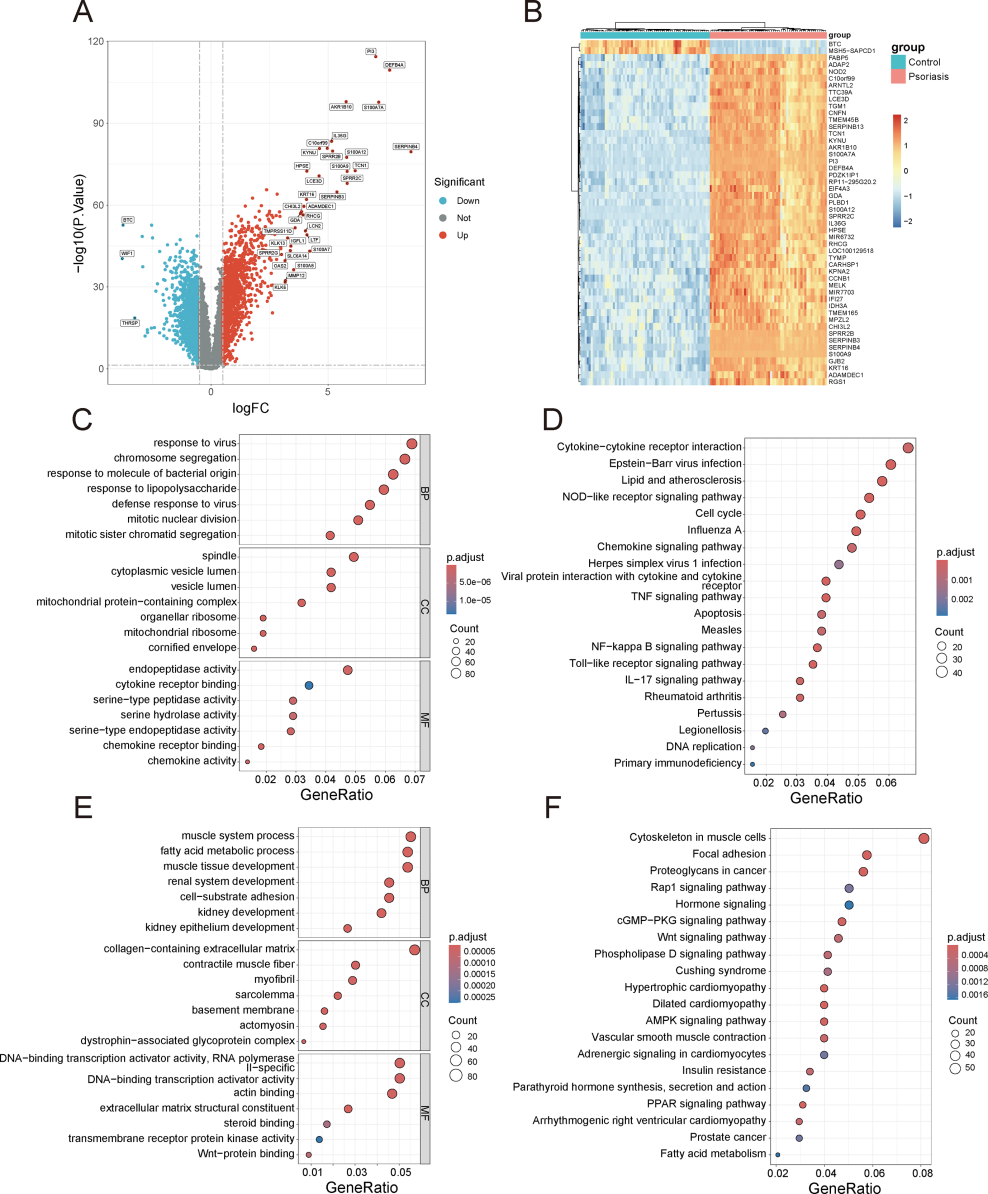
Identification of DEGs in GSE13355 and enrichment analysis of DEGs to elucidate their functions. **(A)** Volcano plot of DEGs, with genes having a p-value less than 0.005 and |logFC| greater than 3 labeled. **(B)** Heatmap of the expression patterns of the top 50 most significantly differentially expressed genes. **(C)** GO analysis of the upregulated genes. **(D)** KEGG analysis of the upregulated genes. **(E)** GO analysis of the downregulated genes. **(F)** KEGG analysis of the downregulated genes.

The study flowchart is presented in **Figure 1**. The volcano plot shows 1,483 upregulated and 1,772 downregulated genes in GSE13355 (**Figure 2A**). Hierarchical clustering highlights distinct expression patterns among the top 50 significant genes (**Figure 2B**). Functional annotation links upregulated genes to cell cycle and immune regulation, with GO analysis showing enrichment in “chromosome segregation” and “chemokine activity” (**Figure 2C**), and KEGG analysis associating them with the “Cell cycle” pathway (**Figure 2D**). Downregulated genes are enriched in GO terms like “collagen-containing extracellular matrix” (**Figure 2E**) and linked to KEGG pathways such as “Focal adhesion” and “Rap1 signaling pathway” (**Figure 2F**).

### 3.2 Identifying psoriasis-associated gene modules and elucidating their functions

**Figure 3:**
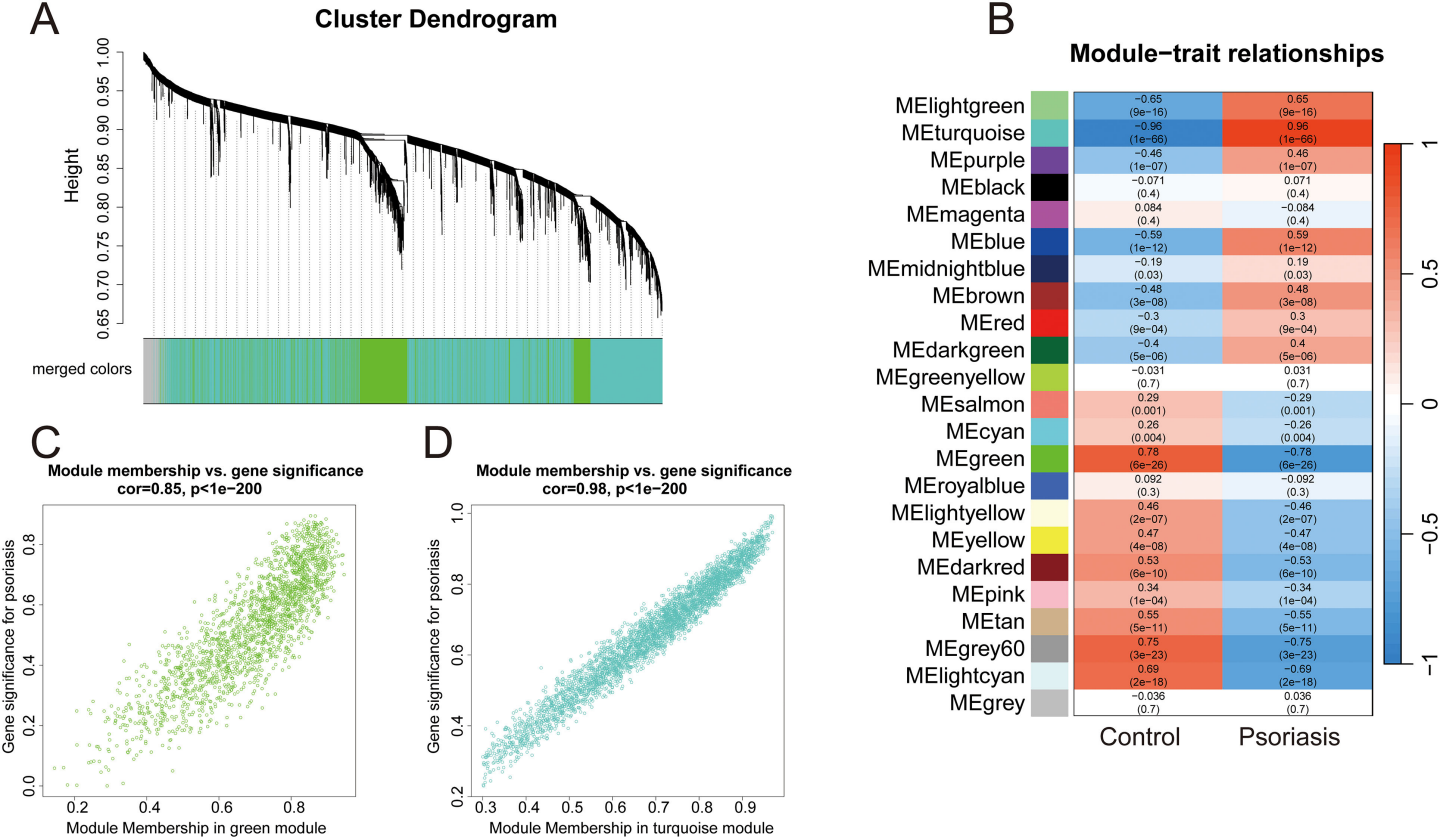
WGCNA. **(A)** Clustering dendrograms of genes. **(B)** Heatmap showing the correlations between gene modules and psoriasis. **(C, D)** Scatter plots of correlations between GS and MM for the turquoise module **(C)** and green module **(D)**.

We conducted WGCNA on GSE13355, removing one outlier with a cutHeight of 76 (**Supplementary Figures S1A, B**). A soft threshold of 6 was selected to ensure a scale-free network (**Supplementary Figure S1C**). We identified 22 gene co-expression modules, with significant correlations to psoriasis (**Figure 3A**). The turquoise module had the highest positive correlation with disease status (r = 0.96, *p* = 1e-66), while the green module had the strongest negative correlation (r = -0.78, *p* = 6e-26) (**Figure 3B**). These findings indicate that the gene clusters may play functional roles in psoriasis development. A strong correlation was found between “Gene significance” (GS) and “module membership” (MM) in the turquoise (0.98) and green (0.85) modules (**Figures 3C, D**), highlighting their importance in psoriasis pathogenesis.

**Figure 4:**
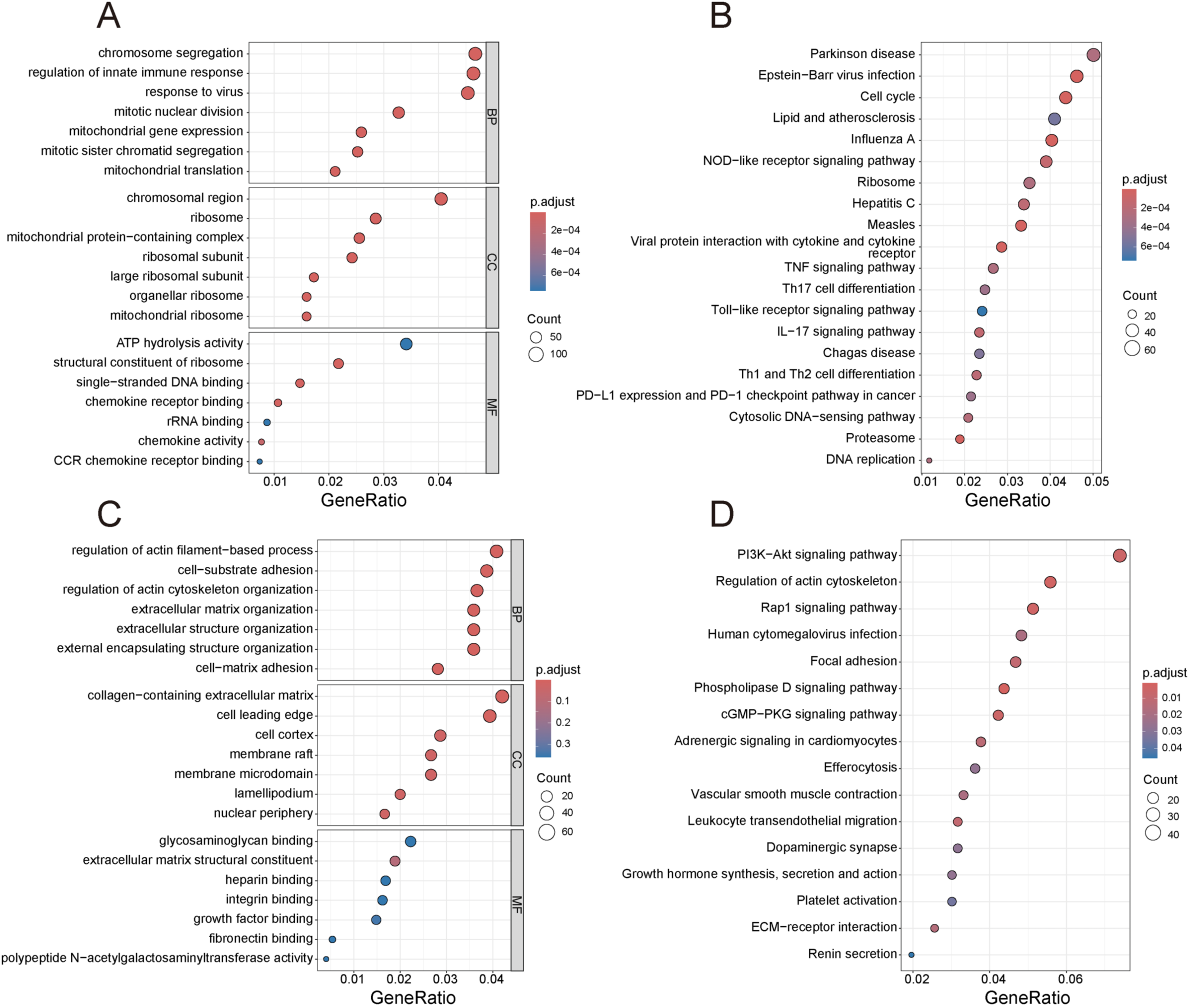
Enrichment analysis of genes within key gene modules to elucidate their functions. **(A)** GO analysis of genes in the turquoise module. **(B)** KEGG analysis of genes in the turquoise module. **(C)** GO analysis of genes in the green module. **(D)** KEGG analysis of genes in the green module.

GO and KEGG analyses of these modules showed that the turquoise module is linked to cell proliferation and immune response modulation. GO analysis revealed enrichment in terms like “mitotic chromosome segregation”, “nuclear division”, and “chemokine-mediated signaling” (**Figure 4A**), while KEGG pathway analysis highlighted “Cell cycle regulation” and “IL-17 mediated inflammatory response” (**Figure 4B**). Conversely, the green module was enriched for GO categories related to cell-matrix interactions and extracellular organization (**Figure 4C**), and KEGG pathways such as “Rap1-dependent signaling cascades” (**Figure 4D**). Notably, the turquoise module’s gene functions aligned with upregulated genes, whereas the green module’s functions matched downregulated genes. The alignment of results across analyses strengthened our study’s credibility and provided new insights into psoriasis pathogenesis.

### 3.3 Identification of key lactylation-related genes and construction of a nomogram model

**Figure 5:**
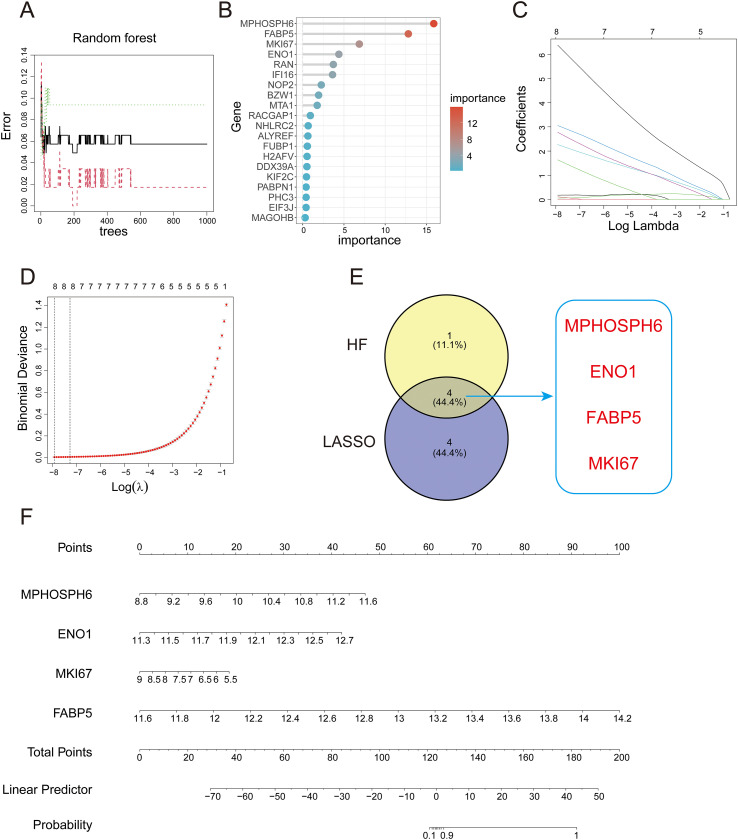
Machine learning-based screening of key diagnostic genes and construction of a nomogram model. **(A, B)** Identifying key diagnostic genes in the GSE13355 dataset via the random forest algorithm. **(C, D)** Identifying key diagnostic genes in the GSE13355 dataset via LASSO regression analysis. **(E)** Intersection of key genes selected by LASSO regression and random forest. **(F)** Nomogram constructed based on the key diagnostic genes.

We observed functional similarities between genes in the turquoise module and upregulated genes, as well as between genes in the green module and downregulated genes. To identify critical genes in psoriasis, we intersected turquoise module genes with upregulated genes and green module genes with downregulated genes, identifying 1,623 key genes. These were further intersected with lactylation-related genes from literature, resulting in 26 lactylation-related key genes (LRKGs), whose proteins can undergo lactylation. We then used Random Forest and LASSO regression to screen for key diagnostic genes from the LRKGs. The Random Forest algorithm identified the top 5 genes by importance (**Figures 5A, B**), while LASSO regression found 8 genes with non-zero coefficients (**Figures 5C, D**). Four genes—MKI67, MPHOSPH6, ENO1, and FABP5—were common to both methods (**Figure 5E**). To assess their combined diagnostic power, a multivariate logistic regression model was created using these four biomarkers, as shown in the nomogram (**Figure 5F**).

### 3.4 Validation of diagnostic efficacy using expression analysis and ROC curves

**Figure 6:**
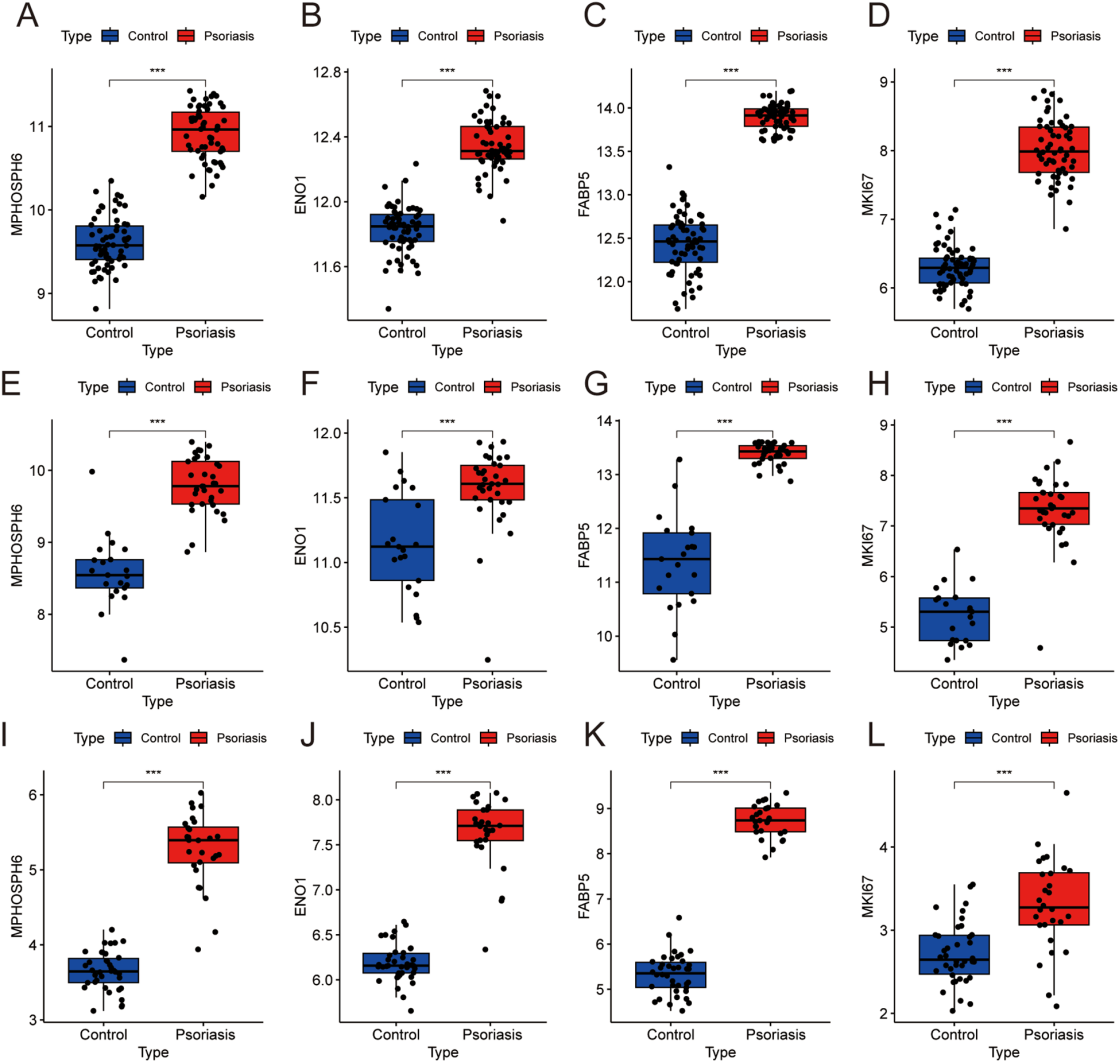
The expression of biomarkers are significantly upregulated across different datasets. **(A-D)** Box plots showing the expression levels of biomarkers in GSE13355. **(E-H)** Box plots showing the expression levels of biomarkers in GSE14905. **(I-L)** Box plots showing the expression levels of biomarkers in GSE121212. ***P < 0.001.

**Figure 7:**
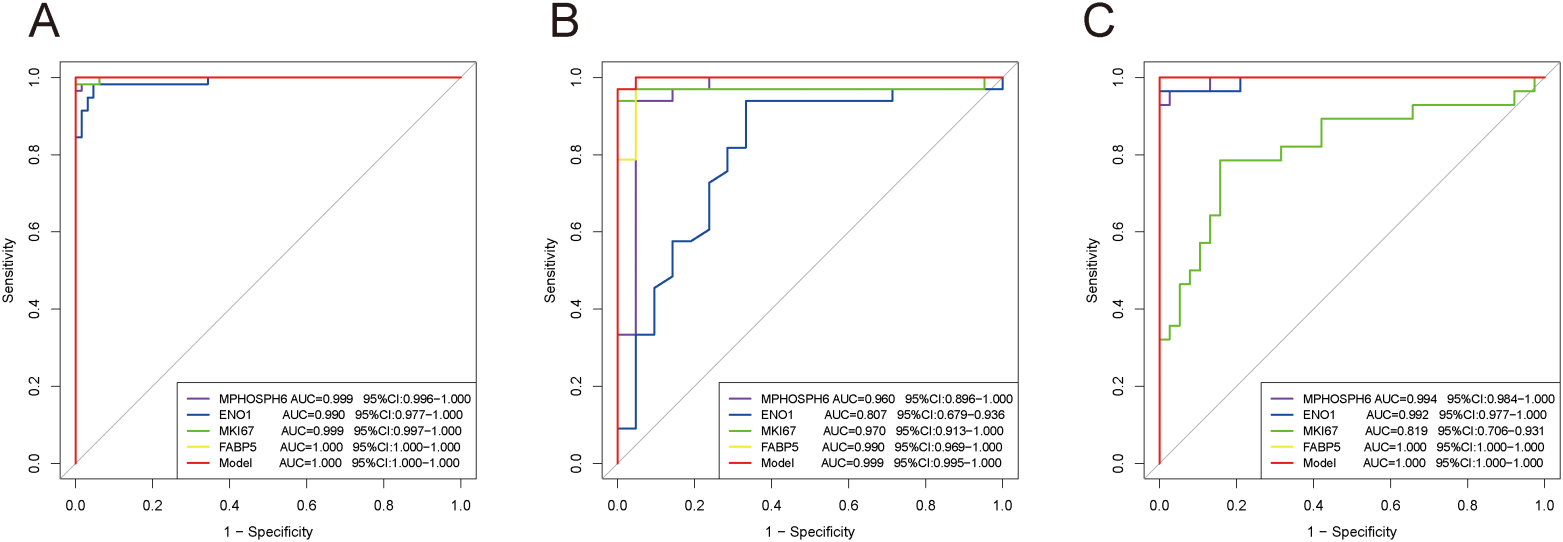
Roc curves of biomarkers in each dataset. **(A)** Roc curves of biomarkers in GSE13355. **(B)** Roc curves of biomarkers in GSE14905. **(C)** Roc curves of biomarkers in GSE121212.

To evaluate the diagnostic potential of four biomarkers, we examined their expression in both training and validation sets. Box plots showed significantly higher levels of MKI67, MPHOSPH6, ENO1, and FABP5 in psoriatic lesions than in healthy controls (**Figure 6**). ROC curve analysis confirmed that each gene, and the combined model, had AUC values over 0.80, indicating strong diagnostic performance (**Figure 7**). The logistic regression model with all four genes achieved an AUC of 0.999 in one dataset and 1.000 in two others (**Figure 7**), underscoring the four-gene signature’s robust diagnostic capability for psoriasis.

### 3.5 Validation of key gene expression in mouse psoriasis models

**Figure 8:**
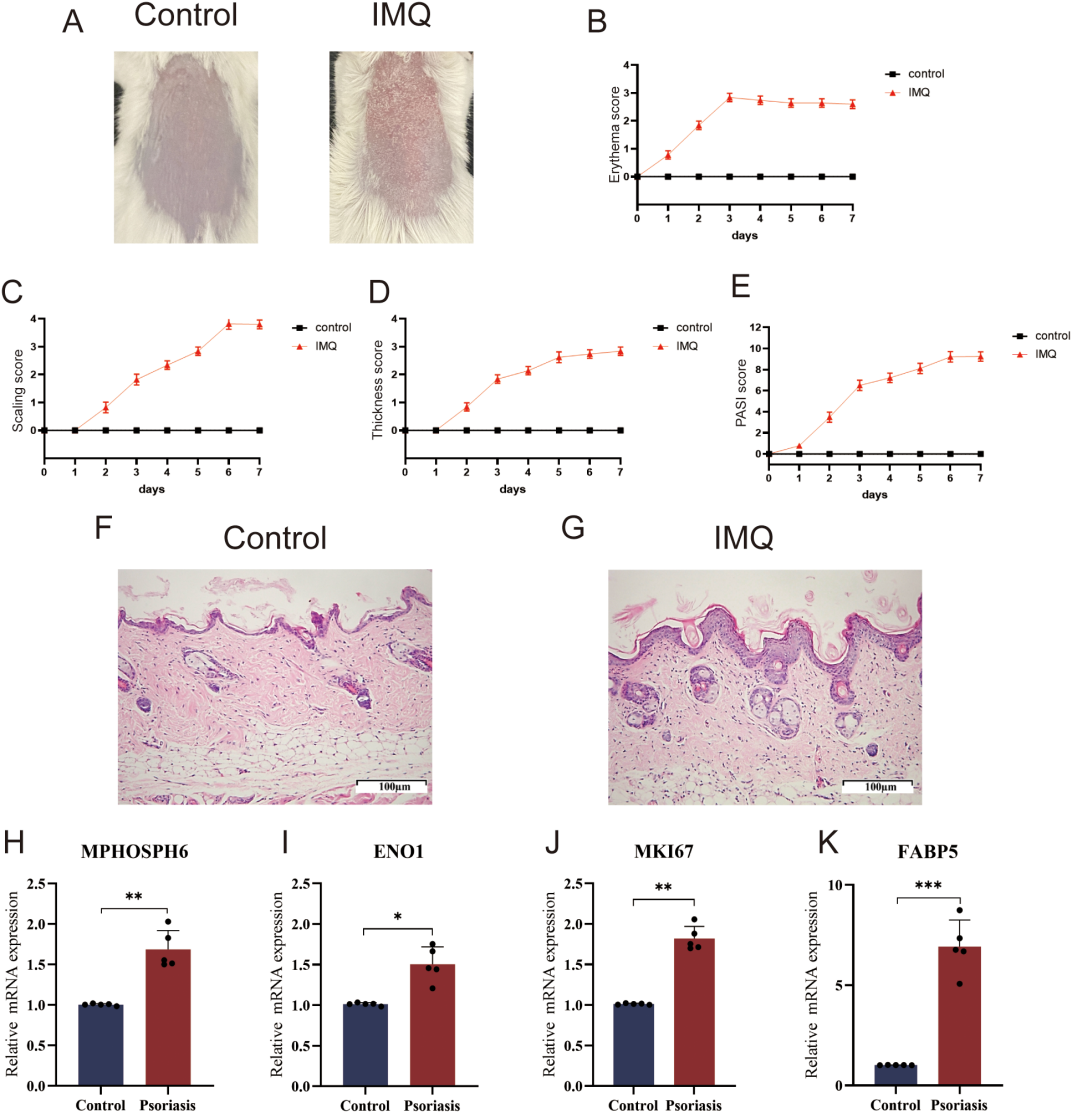
Construction of mouse psoriasis models and validation of biomarkers via RT-qPCR. **(A)** Representative photographs of dorsal skin from a well-established psoriasis mouse model (after 7 days of topical imiquimod application) and a control mouse. **(B-E)** Temporal changes in erythema, scaling, thickness and PASI scores. **(F, G)** HE staining of psoriatic skin and normal skin. **(H-K)** Validation of biomarkers’ expression via RT-qPCR. *P < 0.05, **P < 0.01, ***P < 0.001.

To validate lactylation-related biomarkers, we created imiquimod (IMQ)-induced psoriasis mouse models. IMQ-treated mice showed distinct psoriatic dermatitis features (**Figure 8A**) and higher erythema, scaling, thickness, and PASI scores (**Figures 8B–E**) compared to controls. H&E staining confirmed successful model induction with notable epidermal hyperplasia in IMQ-treated mice (**Figures 8F, G**). RT-qPCR revealed significantly increased expression of MPHOSPH6, ENO1, FABP5, and MKI67 in the lesional skin of IMQ-treated mice versus controls (**Figures 8H–K**).

### 3.6 Immune infiltration analysis

**Figure 9:**
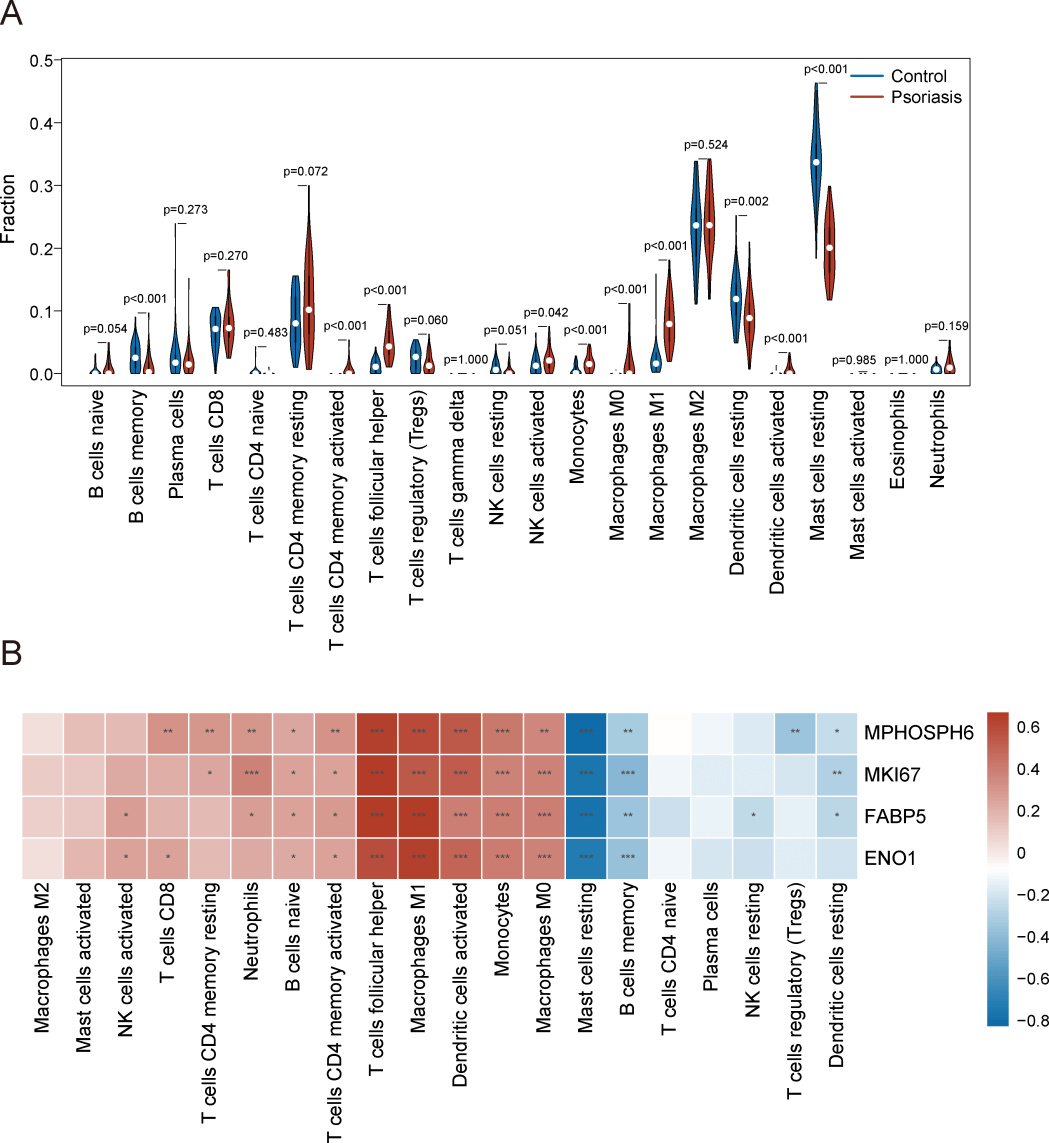
Immune infiltration analysis in psoriasis and correlation between biomarkers and immune cells. **(A)** Violin plots showing the proportion of various immune cells in psoriasis. **(B)** Heatmap showing the correlations between four biomarkers and various immune cells.

To characterize immune cell infiltration in psoriatic skin versus healthy controls, we used the CIBERSORT algorithm to analyze tissue samples from GSE13355. Violin plots showed higher levels of activated CD4^+^ memory T cells, follicular helper T cells, regulatory T cells, activated NK cells, monocytes, M0 macrophages, M1 macrophages, and activated dendritic cells in psoriasis compared to controls (**Figure 9A**). In contrast, memory B cells, resting dendritic cells, and resting mast cells were significantly lower in psoriatic lesions (**Figure 9A**). Additionally, key diagnostic genes were mostly positively correlated with immune cells like naive B cells and follicular helper T cells, while negatively correlated with resting mast cells and memory B cells (**Figure 9B**). These findings highlight a regulatory network connecting gene expression and immune cell activity in psoriasis, indicating possible therapeutic targets for controlling inflammation.

### 3.7 Regulatory networks of miRNAs-genes and TFs-genes and drug target predictions for psoriasis biomarkers

**Figure 10:**
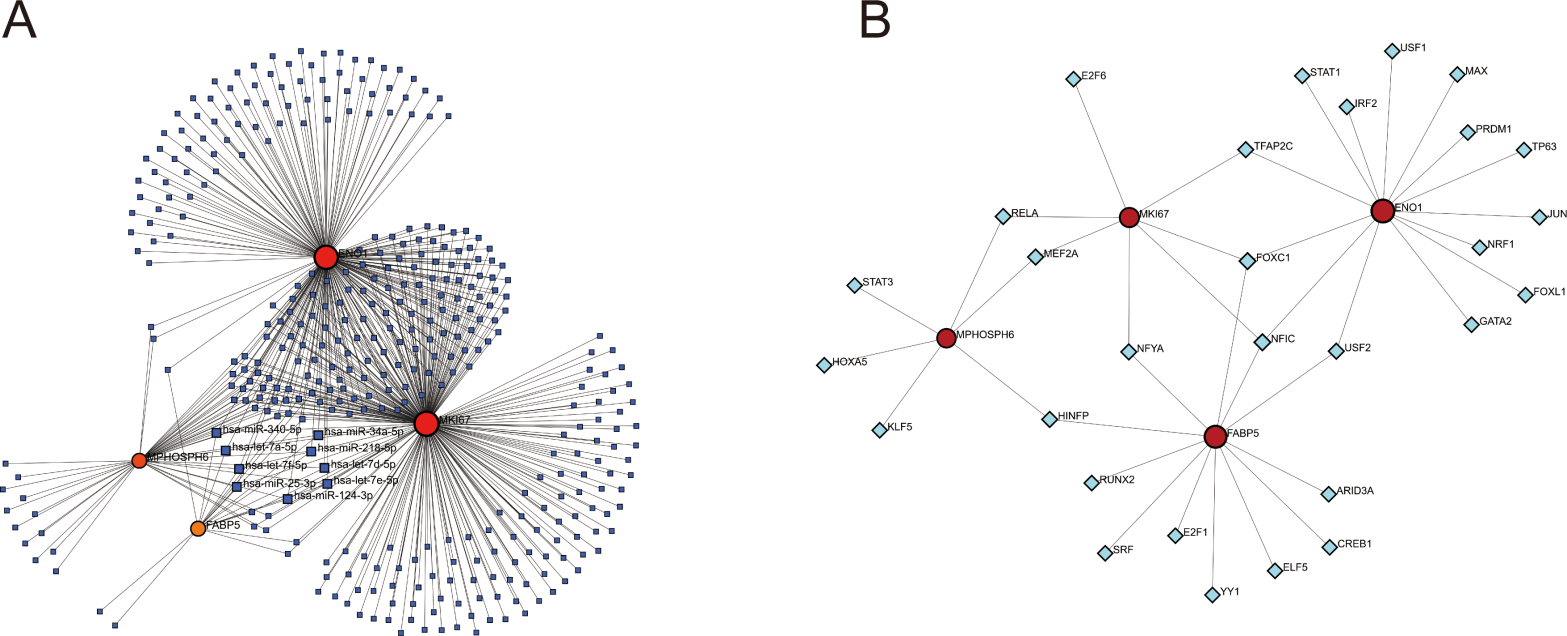
Regulatory networks of biomarkers. **(A)** miRNAs-genes regulatory network. **(B)** TFs-genes regulatory network. In this diagram, red circles represent diagnostic biomarkers, dark blue squares denote microRNAs that interact with the biomarkers, and light blue diamonds indicate transcription factors targeting the biomarkers. The microRNAs targeting all four biomarkers are specifically labeled.

MicroRNAs (miRNAs) and transcription factors (TFs) are crucial in regulating gene expression. To understand the regulatory mechanisms of key diagnostic genes, we created miRNAs-genes and TFs-genes networks using computational predictions. The miRNAs-genes network includes 381 nodes (377 miRNAs and 4 diagnostic genes) and 595 interactions, with MKI67 regulated by 281 miRNAs, ENO1 by 247, MPHOSPH6 by 49, and FABP5 by 18 (**Figure 10A**) (**Supplementary Table 2**). Notably, nine miRNAs regulate all four genes. The TFs-genes network has 33 nodes (29 TFs and 4 genes) and 39 interactions, with ENO1 regulated by 14 TFs, FABP5 by 12, MKI67 by 7, and MPHOSPH6 by 6 (**Figure 10B**) (**Supplementary Table 3**). FOXC1 and NFIC regulate ENO1, FABP5, and MKI67, highlighting their importance as potential key transcription factors for further study. We identified 103 drugs (adjusted *p*-value < 0.05) targeting four biomarkers in the DSigDB database (**Supplementary Table 4**), with the top five most statistically significant drugs listed in **Table 1**.

**Table 1:** Top 5 drugs targeting biomarkers with the smallest p-values in the DSigDB database.

Term	Adjusted P-value	Combined score	Genes
rosiglitazone CTD 00003139	0.0073422088777743725	1482.210166275378	FABP5;ENO1;MKI67
3-Butylidenephthalide CTD 00001227	0.0073422088777743725	3530.6103087148736	ENO1;MKI67
curcumin CTD 00000663	0.0073422088777743725	1061.7092365117326	ENO1;MKI67;MPHOSPH6
Dibenzo[def,p]chrysene CTD 00001899	0.007982776295596843	2227.2163442496094	ENO1;MKI67
clomipramine PC3 DOWN	0.010568152742887001	1626.3490251255419	ENO1;MKI67

### 3.8 Validate the expression profiles and cellular distribution of the biomarkers in single-cell sequencing data

**Figure 11:**
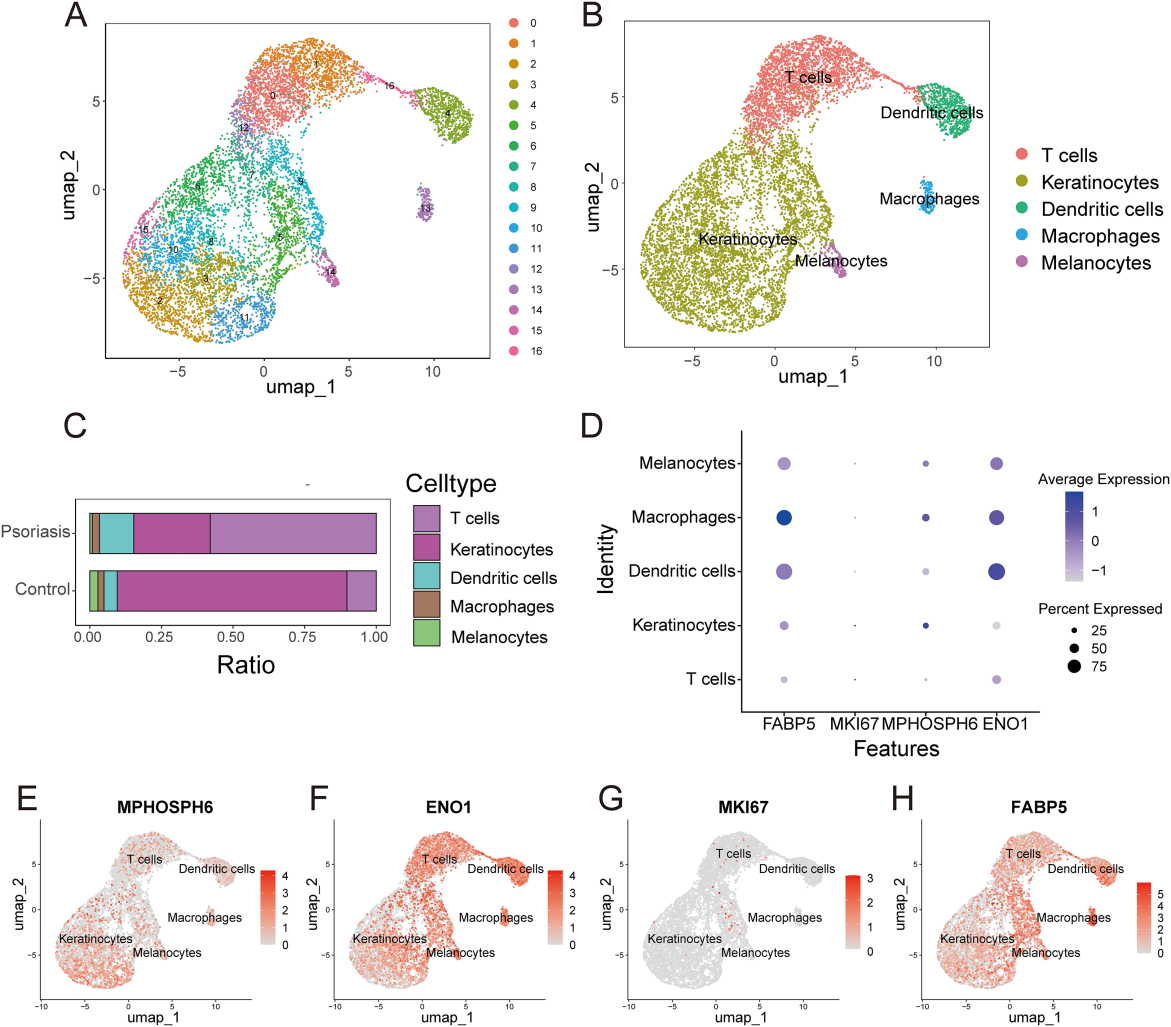
Expression and distribution of biomarkers in single-cell sequencing data. **(A)** UMAP plot of cell clustering. **(B)** UMAP plot of annotated cell populations. **(C)** Proportions of different cell populations in psoriasis and control groups. **(D)** Bubble plot showing the expression proportion and average expression level of biomarkers across different cell populations. **(E-H)** UMAP plots showing the distribution of biomarkers across different cell populations.

The clustering analysis identified 17 distinct cell populations (**Figure 11A**). After SingleR annotation and manual correction, five main cell types were identified: “T cells”, “Keratinocytes”, “Dendritic cells”, “Macrophages”, and “Melanocytes” (**Figure 11B**). Comparative analysis showed a significant increase in T lymphocytes and dendritic cells in lesional skin versus healthy controls, highlighting their roles in psoriasis (**Figure 11C**). This aligns with CIBERSORT algorithm results. A dot plot showed low MKI67 expression across all cell clusters, while other genes varied in expression among cell types (**Figure 11D**). UMAP visualization further detailed the expression profiles and distribution of diagnostic markers across cell clusters (**Figures 11E–H**).

### 3.9 Two-sample Mendelian randomization analysis reveals MPHOSPH6 and ENO1 as risk biomarkers for psoriasis

**Figure 12:**
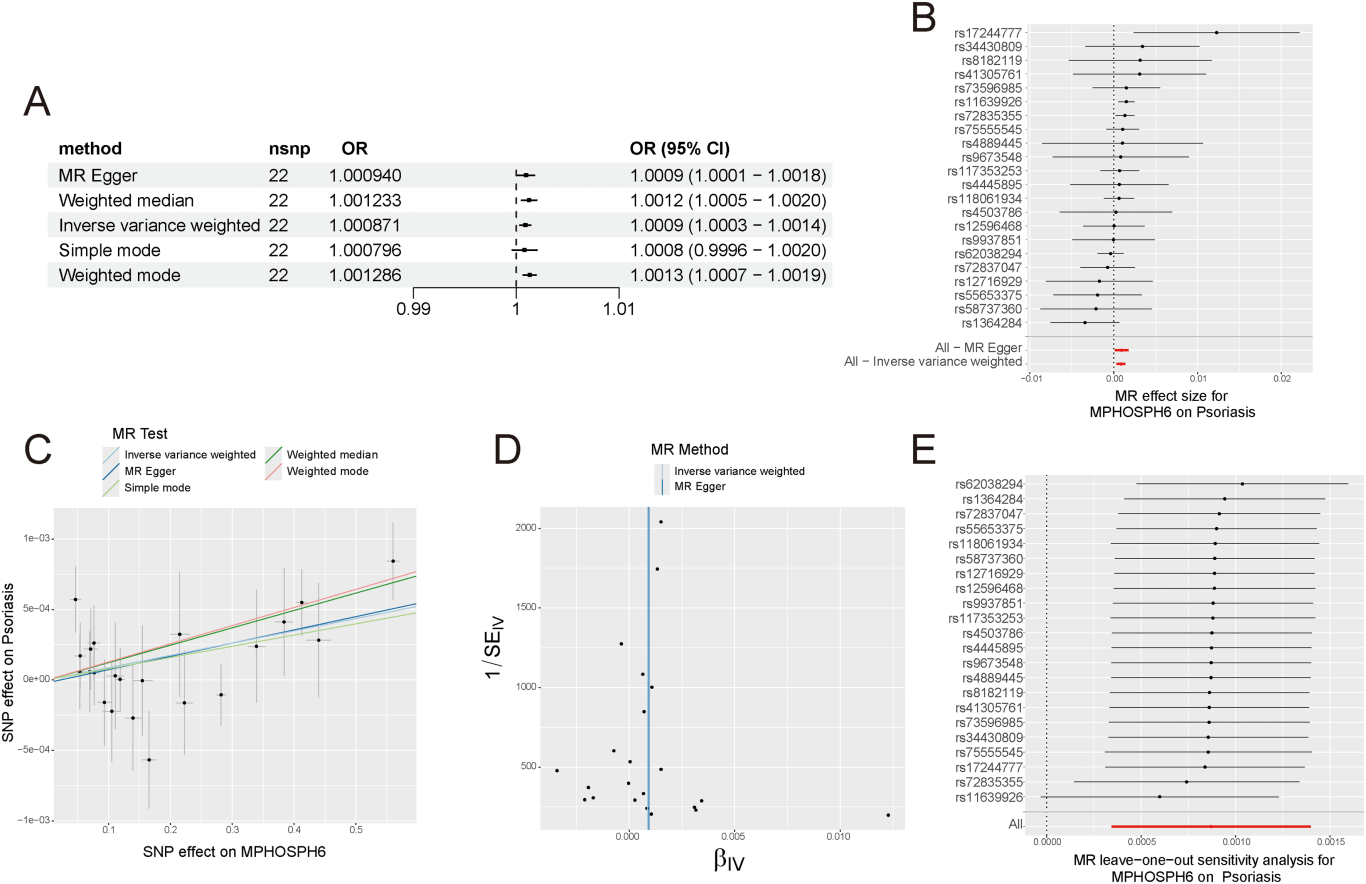
Two-sample mendelian randomization identifies high expression of MPHOSPH6 as a risk factor for psoriasis. **(A)** Causal effects of MPHOSPH6 on psoriasis calculated by five methods. **(B)** Forest plot showing the effects of individual SNPs on psoriasis. **(C)** Scatter plot showing the effects of SNPs on MPHOSPH6 and psoriasis. **(D)** Funnel plot to assess heterogeneity. **(E)** Leave-one-out plot showing the remaining effects after removing individual SNPs.

To examine if key diagnostic genes are causally linked to psoriasis, we performed two-sample Mendelian randomization (TSMR) analysis using eQTL data for MPHOSPH6, ENO1, MKI67, and FABP5 as exposures and psoriasis GWAS data as the outcome. The primary method used was IVW. Our findings indicate a significant causal relationship between MPHOSPH6, ENO1, and psoriasis, with odds ratios (ORs) greater than 1, suggesting their overexpression may increase psoriasis risk. **Figure 12A** shows ORs and *p*-values from five methods assessing MPHOSPH6’s causal link to psoriasis, and **Figure 12B** provides a forest plot of SNP locus effects. A scatter plot shows the effect of each SNP locus on psoriasis and gene expression, with five methods consistently linking MPHOSPH6 to increased psoriasis risk (**Figure 12C**). A funnel plot reveals a symmetrical SNP distribution, suggesting no heterogeneity (**Figure 12D**). A leave-one-out sensitivity analysis confirmed the stability of our findings, as removing any SNP did not significantly change the overall effect estimate, strengthening our conclusions (**Figure 12E**). The **Supplementary Materials** (**Supplementary Figure S2**) detail ENO1 results. A Cochran’s Q test showed no significant heterogeneity (*p* > 0.05), and the MR-Egger regression intercept was near zero with a *p*-value over 0.05, indicating no horizontal pleiotropy. In summary, our TSMR analysis strongly suggests that overexpressing MPHOSPH6 and ENO1 increases psoriasis risk.

### 3.10 SMR analysis further indicates that MPHOSPH6 is a risk biomarker for psoriasis

We conducted SMR analysis, confirming the causal link between MPHOSPH6 and psoriasis with a bSMR of 4.280e-03 and a PSMR of 2.660e-03, both statistically significant. The top SNP was rs34638657, and a PHEIDI value of 0.061 indicated no significant heterogeneity. These results, consistent with TSMR analysis, support a positive causal relationship between higher MPHOSPH6 expression and increased psoriasis risk, strengthening our conclusions.

## 4 Discussion

Psoriasis is a chronic inflammatory skin disorder characterized by dysregulated immune responses and accelerated epidermal proliferation. Understanding the molecular mechanisms underlying psoriasis is crucial for identifying biomarkers and potential therapeutic targets. In this study, we performed a comprehensive analysis of gene expression profiles from the GSE13355 dataset to explore the key genes involved in psoriasis pathogenesis. The differential expression analysis revealed a substantial number of upregulated (1,483) and downregulated (1,772) genes in psoriasis lesions. Functional annotation of the upregulated genes highlighted their enrichment in biological processes such as “chromosome segregation” and “chemokine activity”, underscoring the importance of abnormal cell division and immune cell activation in psoriasis development. The downregulated genes were enriched for processes involved in “collagen-containing extracellular matrix”, pointing to disruptions in tissue homeostasis and extracellular matrix remodeling, which are characteristic features of psoriasis skin lesions. These findings provide a detailed molecular landscape of psoriasis, emphasizing the dual role of immune dysregulation and abnormal cell cycle progression in disease pathogenesis.

In addition to identifying DEGs, we employed WGCNA to uncover gene modules associated with psoriasis. This approach identified 22 gene modules, with two modules, the turquoise and green modules, showing the strongest correlations with psoriasis severity. The turquoise module, positively correlated with disease status (r = 0.96), was strongly associated with processes like cell proliferation and immune response modulation. This module showed enrichment in terms related to “mitotic chromosome segregation”, “nuclear division”, and “chemokine-mediated signaling”, indicating its critical role in regulating keratinocyte proliferation and immune cell recruitment, both of which are central to psoriasis pathology (21, 22). KEGG pathway analysis further reinforced the involvement of the “Cell cycle” and “IL-17 signaling pathway” in the turquoise module, highlighting their potential as therapeutic targets (23, 24). In contrast, the green module, negatively correlated with psoriasis (r = -0.78), was enriched for genes involved in “cell-matrix interactions” and “extracellular organization”, which are key for tissue integrity and homeostasis. These results suggest that psoriasis is driven by a complex interaction between immune system activation, cell cycle dysregulation, and extracellular matrix remodeling (25–27).

In recent years, metabolic reprogramming during inflammatory responses has garnered significant attention (28–30). Studies have revealed elevated glycolysis levels and increased lactate concentrations in psoriasis (31). Lactate is not merely a metabolic waste product; it can also mediate intercellular signal transduction and regulate the immune microenvironment (32, 33). Moreover, lactate can regulate protein function through lactylation modification, exerting a more extensive and profound impact. The intracellular lactylation level of proteins increases with lactate accumulation. It can be inferred that there is widespread protein lactylation in the lesion tissue cells of psoriasis (13). However, the key lactylation - related genes in psoriasis have not been reported yet. To identify critical biomarkers for psoriasis, we intersected genes from the turquoise and green modules with lactylation-related genes, resulting in the identification of 26 key lactylation-related genes (LRKGs). These genes, including MKI67, MPHOSPH6, ENO1, and FABP5, were selected for further investigation due to their potential roles in both psoriasis pathogenesis and lactylation. Using Random Forest and LASSO regression, we identified four genes—MKI67, MPHOSPH6, ENO1, and FABP5—as key diagnostic biomarkers for psoriasis. The diagnostic potential of these biomarkers was validated by analyzing their expression in both the training and validation sets. The results revealed significantly higher expression levels of MKI67, MPHOSPH6, ENO1, and FABP5 in psoriatic lesions compared to healthy controls. ROC curve analysis confirmed the strong diagnostic performance of these biomarkers, with AUC values over 0.80 for each gene, and the combined four-gene model achieving AUC values approaching 1.0, demonstrating its exceptional diagnostic accuracy. To further validate these findings, we used an imiquimod (IMQ)-induced psoriasis mouse model, which confirmed the upregulation of these genes in lesional skin, supporting their relevance as biomarkers for psoriasis.

Immune infiltration, a significant aspect of psoriasis, involves the abnormal activation of the immune system, where T lymphocytes, neutrophils, and macrophages accumulate in the skin (34–36). These cells release inflammatory substances like TNF-α and IFN-γ, leading to excessive skin cell proliferation (37, 38). In this study, immune infiltration analysis revealed significant differences in the immune cell composition of psoriatic skin compared to healthy controls. The CIBERSORT algorithm identified higher levels of various cell types, such as activated CD4^+^ memory T cells, activated NK cells, and M1 macrophages, in psoriasis lesions. Conversely, memory B cells, resting dendritic cells, and resting mast cells were significantly reduced in psoriatic lesions. Notably, the expression of key diagnostic genes, such as MKI67, MPHOSPH6, ENO1, and FABP5, was positively correlated with immune cells like naive B cells and follicular helper T cells, while negatively correlated with resting mast cells and memory B cells. These correlations suggest that these biomarkers may play a role in regulating immune cell activity, which is a hallmark of psoriasis. The interplay between gene expression and immune cell infiltration provides a potential avenue for therapeutic interventions aimed at modulating the immune response in psoriasis.

To understand the regulatory mechanisms controlling these key genes, we constructed miRNA-genes and transcription factor (TF)-genes networks. The miRNA-genes network revealed that MKI67, ENO1, MPHOSPH6, and FABP5 are regulated by a variety of miRNAs, with nine miRNAs regulating all four genes. Notably, previous research has highlighted the downregulation of miR-124-3p in psoriatic lesions and in rIL-22-stimulated HaCaT cells, with its overexpression shown to inhibit cell proliferation and migration capabilities (39). Similarly, miR-218-5p is markedly downregulated in psoriasis, where it acts to suppress keratinocyte proliferation and promote apoptosis by inhibiting IL-36G (40). Additionally, in a case-control investigation, let-7d-5p was discerned to be markedly overexpressed in the blood specimens collected from individuals with psoriasis (41). However, the precise mechanisms through which these miRNAs exert their effects via the regulation of key genes remain to be elucidated and warrant further investigation. The TF-genes network highlighted several important transcription factors, including FOXC1 and NFIC, which regulate multiple biomarkers, suggesting they could be potential targets for modulating gene expression in psoriasis. Additionally, our drug target prediction analysis identified 103 drugs that target the four biomarkers, with the top five most statistically significant drugs offering promising candidates for psoriasis treatment.

Finally, two-sample Mendelian randomization (TSMR) and summary Mendelian randomization (SMR) analyses were conducted to investigate the causal relationship between key diagnostic genes and psoriasis. Our TSMR analysis revealed that MPHOSPH6 and ENO1 are significantly associated with psoriasis risk, with odds ratios greater than 1, indicating that their overexpression may increase the likelihood of developing psoriasis. SMR analysis further confirmed the causal link between MPHOSPH6 and psoriasis, providing additional evidence for its role as a risk biomarker. These findings not only reinforce the potential of MPHOSPH6 and ENO1 as diagnostic biomarkers but also suggest that targeting these genes may offer novel therapeutic strategies for psoriasis.

Although this study validated biomarker expression by constructing mouse psoriasis models, validation in human clinical samples is still necessary to confirm the biological and clinical significance of the identified biomarkers. The imiquimod-induced mouse model, while widely accepted, does not fully capture the complexity and heterogeneity of human psoriasis. The identified biomarkers should be interpreted with appropriate caution until further functional and clinical validation is performed. Future studies involving human psoriatic lesional tissues, longitudinal clinical cohorts, and mechanistic investigations will be essential to fully elucidate the functional roles and clinical applicability of these lactylation-related biomarkers.

## 5 Conclusion

In conclusion, our study provides comprehensive insights into the molecular mechanisms underlying psoriasis by identifying key biomarkers and gene modules associated with disease progression. The diagnostic potential of MKI67, MPHOSPH6, ENO1, and FABP5 offers valuable information for developing more effective diagnostic tools and therapeutic strategies. Future studies focusing on targeting these biomarkers and their associated pathways may lead to more personalized and effective treatments for psoriasis.

## Data Availability

The datasets presented in this study can be found in online repositories. The names of the repository/repositories and accession number(s) can be found in the article/**Supplementary Material**.
