# Supplementary material for: Machine learning meets psoriasis: identifying key lactylation biomarkers as potential targets for diagnosis and therapies
Source: Front Immunol. 2026 Mar 13;17:1791693. doi: 10.3389/fimmu.2026.1791693 (PMC13021890; doi:10.3389/fimmu.2026.1791693)
Supplement: Supplementary file 1 [file Table1.docx]

**Supplementary materials:**


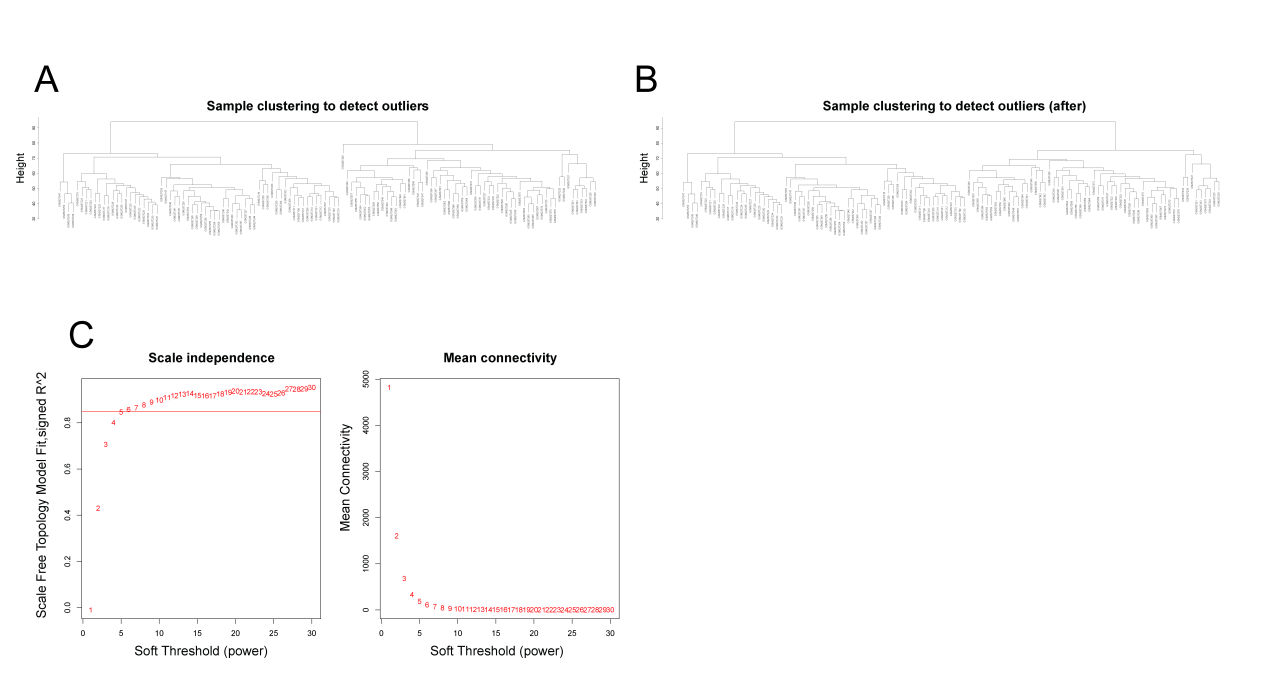


**Supplementary Figure 1 WGCNA analysis on GSE13355.** (A) Sample clustering before removing outliers. (B) Sample clustering after removing outliers. (C) Selection of an appropriate soft threshold.


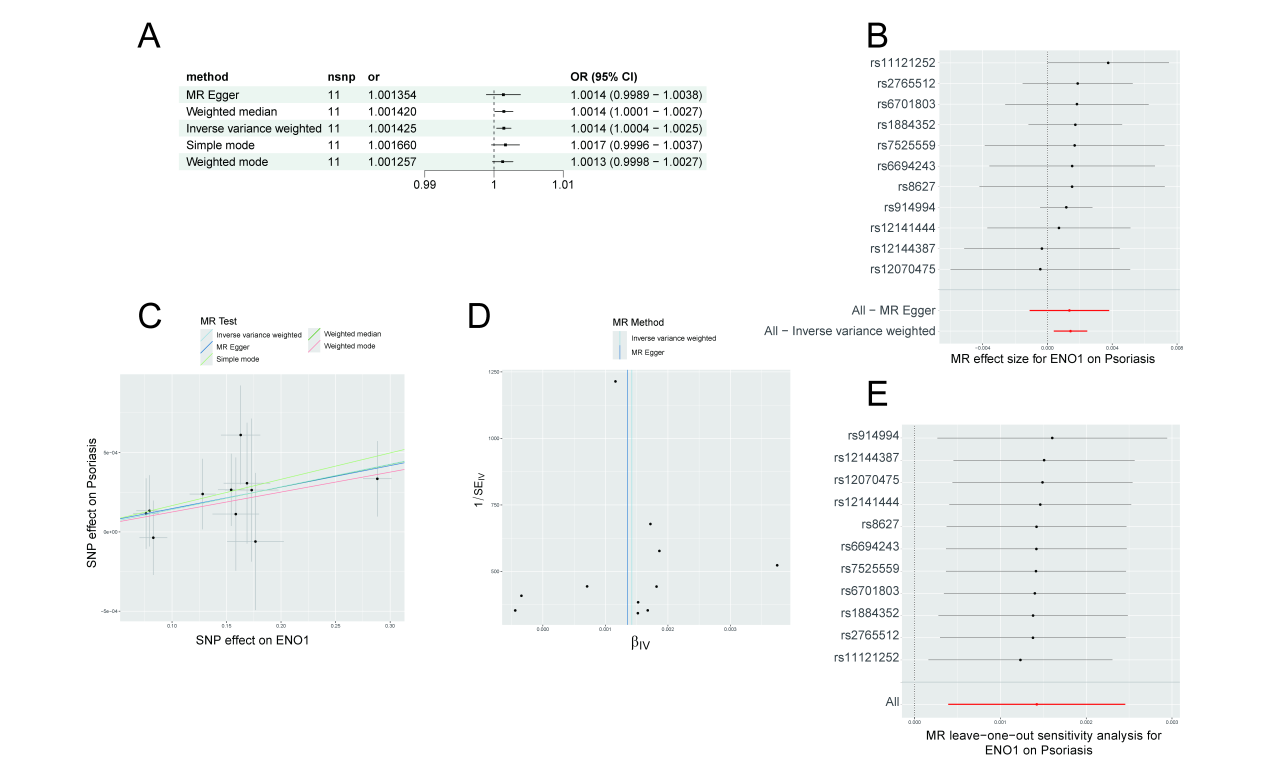


**Supplementary Figure 2 Two-sample mendelian randomization identifies high expression of ENO1 as a risk factor for psoriasis.** (A) Causal effects of ENO1 on psoriasis calculated by five methods. (B) Forest plot. (C) Scatter plot. (D) Funnel plot. (E) Leave-one-out plot.

Supplementary Table 1: PCR primer sequences.

| **Gene** | **Forward** | **Reverse** |
| --- | --- | --- |
| MPHOSPH6 | GAGCGCAAGACTAAGTTATCCAA | TCTCCTTCAACTCCGGCAAAT |
| ENO1 | TGCGTCCACTGGCATCTAC | CAGAGCAGGCGCAATAGTTTTA |
| MKI67 | ATCATTGACCGCTCCTTTAGGT | GCTCGCCTTGATGGTTCCT |
| FABP5 | TGAAAGAGCTAGGAGTAGGACTG | CTCTCGGTTTTGACCGTGATG |
| GAPDH | CAGGAGGCATTGCTGATGAT | GAAGGCTGGGGCTCATTT |

Supplementary Table 2: Details of the miRNAs-genes regulatory network.

| **Id** | **Label** | **Degree** | **Betweenness** | **Expression** |
| --- | --- | --- | --- | --- |
| 4288 | MKI67 | 281 | 44871.14 | 0 |
| 2023 | ENO1 | 247 | 35352.12 | 0 |
| 10200 | MPHOSPH6 | 49 | 4853.32 | 0 |
| 2171 | FABP5 | 18 | 843.41 | 0 |
| MIMAT0000062 | hsa-let-7a-5p | 4 | 267.93 | 0 |
| MIMAT0000065 | hsa-let-7d-5p | 4 | 267.93 | 0 |
| MIMAT0000066 | hsa-let-7e-5p | 4 | 267.93 | 0 |
| MIMAT0000067 | hsa-let-7f-5p | 4 | 267.93 | 0 |
| MIMAT0000081 | hsa-miR-25-3p | 4 | 267.93 | 0 |
| MIMAT0000255 | hsa-miR-34a-5p | 4 | 267.93 | 0 |
| MIMAT0000275 | hsa-miR-218-5p | 4 | 267.93 | 0 |
| MIMAT0000422 | hsa-miR-124-3p | 4 | 267.93 | 0 |
| MIMAT0004692 | hsa-miR-340-5p | 4 | 267.93 | 0 |
| MIMAT0000063 | hsa-let-7b-5p | 3 | 185.51 | 0 |
| MIMAT0000064 | hsa-let-7c-5p | 3 | 185.51 | 0 |
| MIMAT0000068 | hsa-miR-15a-5p | 3 | 185.51 | 0 |
| MIMAT0000069 | hsa-miR-16-5p | 3 | 185.51 | 0 |
| MIMAT0000073 | hsa-miR-19a-3p | 3 | 185.51 | 0 |
| MIMAT0000074 | hsa-miR-19b-3p | 3 | 185.51 | 0 |
| MIMAT0000087 | hsa-miR-30a-5p | 3 | 185.51 | 0 |
| MIMAT0000096 | hsa-miR-98-5p | 3 | 185.51 | 0 |
| MIMAT0000101 | hsa-miR-103a-3p | 3 | 185.51 | 0 |
| MIMAT0000256 | hsa-miR-181a-5p | 3 | 185.51 | 0 |
| MIMAT0000259 | hsa-miR-182-5p | 3 | 185.51 | 0 |
| MIMAT0000414 | hsa-let-7g-5p | 3 | 185.51 | 0 |
| MIMAT0000415 | hsa-let-7i-5p | 3 | 185.51 | 0 |
| MIMAT0000417 | hsa-miR-15b-5p | 3 | 185.51 | 0 |
| MIMAT0000440 | hsa-miR-191-5p | 3 | 185.51 | 0 |
| MIMAT0000455 | hsa-miR-185-5p | 3 | 185.51 | 0 |
| MIMAT0000460 | hsa-miR-194-5p | 3 | 185.51 | 0 |
| MIMAT0000732 | hsa-miR-378a-3p | 3 | 185.51 | 0 |
| MIMAT0001341 | hsa-miR-424-5p | 3 | 185.51 | 0 |
| MIMAT0001541 | hsa-miR-449a | 3 | 185.51 | 0 |
| MIMAT0002874 | hsa-miR-503-5p | 3 | 185.51 | 0 |
| MIMAT0003218 | hsa-miR-92b-3p | 3 | 185.51 | 0 |
| MIMAT0000098 | hsa-miR-100-5p | 3 | 132.82 | 0 |
| MIMAT0000267 | hsa-miR-210-3p | 3 | 132.82 | 0 |
| MIMAT0004501 | hsa-miR-27a-5p | 3 | 132.82 | 0 |
| MIMAT0004605 | hsa-miR-129-2-3p | 3 | 115.11 | 0 |
| MIMAT0000264 | hsa-miR-203a-3p | 2 | 68.48 | 0 |
| MIMAT0000416 | hsa-miR-1-3p | 2 | 68.48 | 0 |
| MIMAT0003249 | hsa-miR-584-5p | 2 | 68.48 | 0 |
| MIMAT0000258 | hsa-miR-181c-5p | 2 | 58.97 | 0 |
| MIMAT0002888 | hsa-miR-532-5p | 2 | 58.97 | 0 |
| MIMAT0000070 | hsa-miR-17-5p | 2 | 58.07 | 0 |
| MIMAT0000071 | hsa-miR-17-3p | 2 | 58.07 | 0 |
| MIMAT0000072 | hsa-miR-18a-5p | 2 | 58.07 | 0 |
| MIMAT0000075 | hsa-miR-20a-5p | 2 | 58.07 | 0 |
| MIMAT0000076 | hsa-miR-21-5p | 2 | 58.07 | 0 |
| MIMAT0000077 | hsa-miR-22-3p | 2 | 58.07 | 0 |
| MIMAT0000078 | hsa-miR-23a-3p | 2 | 58.07 | 0 |
| MIMAT0000080 | hsa-miR-24-3p | 2 | 58.07 | 0 |
| MIMAT0000082 | hsa-miR-26a-5p | 2 | 58.07 | 0 |
| MIMAT0000084 | hsa-miR-27a-3p | 2 | 58.07 | 0 |
| MIMAT0000085 | hsa-miR-28-5p | 2 | 58.07 | 0 |
| MIMAT0000086 | hsa-miR-29a-3p | 2 | 58.07 | 0 |
| MIMAT0000088 | hsa-miR-30a-3p | 2 | 58.07 | 0 |
| MIMAT0000089 | hsa-miR-31-5p | 2 | 58.07 | 0 |
| MIMAT0000091 | hsa-miR-33a-5p | 2 | 58.07 | 0 |
| MIMAT0000092 | hsa-miR-92a-3p | 2 | 58.07 | 0 |
| MIMAT0000093 | hsa-miR-93-5p | 2 | 58.07 | 0 |
| MIMAT0000095 | hsa-miR-96-5p | 2 | 58.07 | 0 |
| MIMAT0000100 | hsa-miR-29b-3p | 2 | 58.07 | 0 |
| MIMAT0000103 | hsa-miR-106a-5p | 2 | 58.07 | 0 |
| MIMAT0000104 | hsa-miR-107 | 2 | 58.07 | 0 |
| MIMAT0000226 | hsa-miR-196a-5p | 2 | 58.07 | 0 |
| MIMAT0000243 | hsa-miR-148a-3p | 2 | 58.07 | 0 |
| MIMAT0000245 | hsa-miR-30d-5p | 2 | 58.07 | 0 |
| MIMAT0000250 | hsa-miR-139-5p | 2 | 58.07 | 0 |
| MIMAT0000251 | hsa-miR-147a | 2 | 58.07 | 0 |
| MIMAT0000252 | hsa-miR-7-5p | 2 | 58.07 | 0 |
| MIMAT0000257 | hsa-miR-181b-5p | 2 | 58.07 | 0 |
| MIMAT0000260 | hsa-miR-182-3p | 2 | 58.07 | 0 |
| MIMAT0000261 | hsa-miR-183-5p | 2 | 58.07 | 0 |
| MIMAT0000266 | hsa-miR-205-5p | 2 | 58.07 | 0 |
| MIMAT0000418 | hsa-miR-23b-3p | 2 | 58.07 | 0 |
| MIMAT0000419 | hsa-miR-27b-3p | 2 | 58.07 | 0 |
| MIMAT0000420 | hsa-miR-30b-5p | 2 | 58.07 | 0 |
| MIMAT0000423 | hsa-miR-125b-5p | 2 | 58.07 | 0 |
| MIMAT0000424 | hsa-miR-128-3p | 2 | 58.07 | 0 |
| MIMAT0000425 | hsa-miR-130a-3p | 2 | 58.07 | 0 |
| MIMAT0000427 | hsa-miR-133a-3p | 2 | 58.07 | 0 |
| MIMAT0000437 | hsa-miR-145-5p | 2 | 58.07 | 0 |
| MIMAT0000438 | hsa-miR-152-3p | 2 | 58.07 | 0 |
| MIMAT0000441 | hsa-miR-9-5p | 2 | 58.07 | 0 |
| MIMAT0000443 | hsa-miR-125a-5p | 2 | 58.07 | 0 |
| MIMAT0000449 | hsa-miR-146a-5p | 2 | 58.07 | 0 |
| MIMAT0000450 | hsa-miR-149-5p | 2 | 58.07 | 0 |
| MIMAT0000457 | hsa-miR-188-5p | 2 | 58.07 | 0 |
| MIMAT0000461 | hsa-miR-195-5p | 2 | 58.07 | 0 |
| MIMAT0000680 | hsa-miR-106b-5p | 2 | 58.07 | 0 |
| MIMAT0000681 | hsa-miR-29c-3p | 2 | 58.07 | 0 |
| MIMAT0000688 | hsa-miR-301a-3p | 2 | 58.07 | 0 |
| MIMAT0000691 | hsa-miR-130b-3p | 2 | 58.07 | 0 |
| MIMAT0000693 | hsa-miR-30e-3p | 2 | 58.07 | 0 |
| MIMAT0000703 | hsa-miR-361-5p | 2 | 58.07 | 0 |
| MIMAT0000727 | hsa-miR-374a-5p | 2 | 58.07 | 0 |
| MIMAT0000750 | hsa-miR-340-3p | 2 | 58.07 | 0 |
| MIMAT0000758 | hsa-miR-135b-5p | 2 | 58.07 | 0 |
| MIMAT0000759 | hsa-miR-148b-3p | 2 | 58.07 | 0 |
| MIMAT0000762 | hsa-miR-324-3p | 2 | 58.07 | 0 |
| MIMAT0000763 | hsa-miR-338-3p | 2 | 58.07 | 0 |
| MIMAT0000765 | hsa-miR-335-5p | 2 | 58.07 | 0 |
| MIMAT0001080 | hsa-miR-196b-5p | 2 | 58.07 | 0 |
| MIMAT0001340 | hsa-miR-423-3p | 2 | 58.07 | 0 |
| MIMAT0001413 | hsa-miR-20b-5p | 2 | 58.07 | 0 |
| MIMAT0001620 | hsa-miR-200a-5p | 2 | 58.07 | 0 |
| MIMAT0002174 | hsa-miR-484 | 2 | 58.07 | 0 |
| MIMAT0002809 | hsa-miR-146b-5p | 2 | 58.07 | 0 |
| MIMAT0002821 | hsa-miR-181d-5p | 2 | 58.07 | 0 |
| MIMAT0002830 | hsa-miR-520f-3p | 2 | 58.07 | 0 |
| MIMAT0002835 | hsa-miR-526b-5p | 2 | 58.07 | 0 |
| MIMAT0002871 | hsa-miR-500a-3p | 2 | 58.07 | 0 |
| MIMAT0002876 | hsa-miR-505-3p | 2 | 58.07 | 0 |
| MIMAT0003150 | hsa-miR-455-5p | 2 | 58.07 | 0 |
| MIMAT0003294 | hsa-miR-625-5p | 2 | 58.07 | 0 |
| MIMAT0003301 | hsa-miR-33b-5p | 2 | 58.07 | 0 |
| MIMAT0003340 | hsa-miR-542-5p | 2 | 58.07 | 0 |
| MIMAT0003393 | hsa-miR-425-5p | 2 | 58.07 | 0 |
| MIMAT0003880 | hsa-miR-671-5p | 2 | 58.07 | 0 |
| MIMAT0003885 | hsa-miR-454-3p | 2 | 58.07 | 0 |
| MIMAT0003886 | hsa-miR-769-5p | 2 | 58.07 | 0 |
| MIMAT0003888 | hsa-miR-766-3p | 2 | 58.07 | 0 |
| MIMAT0004487 | hsa-let-7f-2-3p | 2 | 58.07 | 0 |
| MIMAT0004488 | hsa-miR-15a-3p | 2 | 58.07 | 0 |
| MIMAT0004490 | hsa-miR-19a-5p | 2 | 58.07 | 0 |
| MIMAT0004491 | hsa-miR-19b-1-5p | 2 | 58.07 | 0 |
| MIMAT0004495 | hsa-miR-22-5p | 2 | 58.07 | 0 |
| MIMAT0004509 | hsa-miR-93-3p | 2 | 58.07 | 0 |
| MIMAT0004512 | hsa-miR-100-3p | 2 | 58.07 | 0 |
| MIMAT0004550 | hsa-miR-30c-2-3p | 2 | 58.07 | 0 |
| MIMAT0004558 | hsa-miR-181a-2-3p | 2 | 58.07 | 0 |
| MIMAT0004568 | hsa-miR-221-5p | 2 | 58.07 | 0 |
| MIMAT0004571 | hsa-miR-200b-5p | 2 | 58.07 | 0 |
| MIMAT0004586 | hsa-miR-15b-3p | 2 | 58.07 | 0 |
| MIMAT0004589 | hsa-miR-30b-3p | 2 | 58.07 | 0 |
| MIMAT0004597 | hsa-miR-140-3p | 2 | 58.07 | 0 |
| MIMAT0004598 | hsa-miR-141-5p | 2 | 58.07 | 0 |
| MIMAT0004601 | hsa-miR-145-3p | 2 | 58.07 | 0 |
| MIMAT0004602 | hsa-miR-125a-3p | 2 | 58.07 | 0 |
| MIMAT0004603 | hsa-miR-125b-2-3p | 2 | 58.07 | 0 |
| MIMAT0004672 | hsa-miR-106b-3p | 2 | 58.07 | 0 |
| MIMAT0004680 | hsa-miR-130b-5p | 2 | 58.07 | 0 |
| MIMAT0004682 | hsa-miR-361-3p | 2 | 58.07 | 0 |
| MIMAT0004688 | hsa-miR-374a-3p | 2 | 58.07 | 0 |
| MIMAT0004697 | hsa-miR-151a-5p | 2 | 58.07 | 0 |
| MIMAT0004699 | hsa-miR-148b-5p | 2 | 58.07 | 0 |
| MIMAT0004700 | hsa-miR-331-5p | 2 | 58.07 | 0 |
| MIMAT0004748 | hsa-miR-423-5p | 2 | 58.07 | 0 |
| MIMAT0004762 | hsa-miR-486-3p | 2 | 58.07 | 0 |
| MIMAT0004767 | hsa-miR-193b-5p | 2 | 58.07 | 0 |
| MIMAT0004799 | hsa-miR-589-5p | 2 | 58.07 | 0 |
| MIMAT0004811 | hsa-miR-33b-3p | 2 | 58.07 | 0 |
| MIMAT0004909 | hsa-miR-450b-5p | 2 | 58.07 | 0 |
| MIMAT0004945 | hsa-miR-744-5p | 2 | 58.07 | 0 |
| MIMAT0004985 | hsa-miR-942-5p | 2 | 58.07 | 0 |
| MIMAT0005792 | hsa-miR-320b | 2 | 58.07 | 0 |
| MIMAT0005793 | hsa-miR-320c | 2 | 58.07 | 0 |
| MIMAT0005797 | hsa-miR-1301-3p | 2 | 58.07 | 0 |
| MIMAT0005876 | hsa-miR-1285-3p | 2 | 58.07 | 0 |
| MIMAT0006764 | hsa-miR-320d | 2 | 58.07 | 0 |
| MIMAT0007885 | hsa-miR-1911-5p | 2 | 58.07 | 0 |
| MIMAT0015031 | hsa-miR-3157-5p | 2 | 58.07 | 0 |
| MIMAT0015064 | hsa-miR-3184-5p | 2 | 58.07 | 0 |
| MIMAT0015085 | hsa-miR-3200-3p | 2 | 58.07 | 0 |
| MIMAT0016847 | hsa-miR-378c | 2 | 58.07 | 0 |
| MIMAT0019058 | hsa-miR-4521 | 2 | 58.07 | 0 |
| MIMAT0019761 | hsa-miR-4677-3p | 2 | 58.07 | 0 |
| MIMAT0019813 | hsa-miR-203b-5p | 2 | 58.07 | 0 |
| MIMAT0022706 | hsa-miR-561-5p | 2 | 58.07 | 0 |
| MIMAT0022731 | hsa-miR-3184-3p | 2 | 58.07 | 0 |
| MIMAT0031177 | hsa-miR-7974 | 2 | 58.07 | 0 |
| MIMAT0004928 | hsa-miR-147b-3p | 2 | 58.07 | 0 |
| MIMAT0000510 | hsa-miR-320a-3p | 2 | 58.07 | 0 |
| MIMAT0000728 | hsa-miR-375-3p | 2 | 58.07 | 0 |
| MIMAT0000254 | hsa-miR-10b-5p | 2 | 38.96 | 0 |
| MIMAT0004494 | hsa-miR-21-3p | 2 | 38.96 | 0 |
| MIMAT0000097 | hsa-miR-99a-5p | 2 | 35.79 | 0 |
| MIMAT0000083 | hsa-miR-26b-5p | 1 | 0 | 0 |
| MIMAT0000094 | hsa-miR-95-3p | 1 | 0 | 0 |
| MIMAT0000099 | hsa-miR-101-3p | 1 | 0 | 0 |
| MIMAT0000222 | hsa-miR-192-5p | 1 | 0 | 0 |
| MIMAT0000227 | hsa-miR-197-3p | 1 | 0 | 0 |
| MIMAT0000232 | hsa-miR-199a-3p | 1 | 0 | 0 |
| MIMAT0000244 | hsa-miR-30c-5p | 1 | 0 | 0 |
| MIMAT0000253 | hsa-miR-10a-5p | 1 | 0 | 0 |
| MIMAT0000265 | hsa-miR-204-5p | 1 | 0 | 0 |
| MIMAT0000269 | hsa-miR-212-3p | 1 | 0 | 0 |
| MIMAT0000278 | hsa-miR-221-3p | 1 | 0 | 0 |
| MIMAT0000279 | hsa-miR-222-3p | 1 | 0 | 0 |
| MIMAT0000280 | hsa-miR-223-3p | 1 | 0 | 0 |
| MIMAT0000281 | hsa-miR-224-5p | 1 | 0 | 0 |
| MIMAT0000318 | hsa-miR-200b-3p | 1 | 0 | 0 |
| MIMAT0000426 | hsa-miR-132-3p | 1 | 0 | 0 |
| MIMAT0000430 | hsa-miR-138-5p | 1 | 0 | 0 |
| MIMAT0000431 | hsa-miR-140-5p | 1 | 0 | 0 |
| MIMAT0000432 | hsa-miR-141-3p | 1 | 0 | 0 |
| MIMAT0000434 | hsa-miR-142-3p | 1 | 0 | 0 |
| MIMAT0000444 | hsa-miR-126-5p | 1 | 0 | 0 |
| MIMAT0000445 | hsa-miR-126-3p | 1 | 0 | 0 |
| MIMAT0000451 | hsa-miR-150-5p | 1 | 0 | 0 |
| MIMAT0000456 | hsa-miR-186-5p | 1 | 0 | 0 |
| MIMAT0000459 | hsa-miR-193a-3p | 1 | 0 | 0 |
| MIMAT0000646 | hsa-miR-155-5p | 1 | 0 | 0 |
| MIMAT0000682 | hsa-miR-200a-3p | 1 | 0 | 0 |
| MIMAT0000684 | hsa-miR-302a-3p | 1 | 0 | 0 |
| MIMAT0000686 | hsa-miR-34c-5p | 1 | 0 | 0 |
| MIMAT0000689 | hsa-miR-99b-5p | 1 | 0 | 0 |
| MIMAT0000690 | hsa-miR-296-5p | 1 | 0 | 0 |
| MIMAT0000692 | hsa-miR-30e-5p | 1 | 0 | 0 |
| MIMAT0000705 | hsa-miR-362-5p | 1 | 0 | 0 |
| MIMAT0000707 | hsa-miR-363-3p | 1 | 0 | 0 |
| MIMAT0000718 | hsa-miR-302d-3p | 1 | 0 | 0 |
| MIMAT0000721 | hsa-miR-369-3p | 1 | 0 | 0 |
| MIMAT0000722 | hsa-miR-370-3p | 1 | 0 | 0 |
| MIMAT0000731 | hsa-miR-378a-5p | 1 | 0 | 0 |
| MIMAT0000733 | hsa-miR-379-5p | 1 | 0 | 0 |
| MIMAT0000734 | hsa-miR-380-5p | 1 | 0 | 0 |
| MIMAT0000764 | hsa-miR-339-5p | 1 | 0 | 0 |
| MIMAT0000772 | hsa-miR-345-5p | 1 | 0 | 0 |
| MIMAT0001343 | hsa-miR-425-3p | 1 | 0 | 0 |
| MIMAT0001412 | hsa-miR-18b-5p | 1 | 0 | 0 |
| MIMAT0001545 | hsa-miR-450a-5p | 1 | 0 | 0 |
| MIMAT0001627 | hsa-miR-433-3p | 1 | 0 | 0 |
| MIMAT0002173 | hsa-miR-483-3p | 1 | 0 | 0 |
| MIMAT0002175 | hsa-miR-485-5p | 1 | 0 | 0 |
| MIMAT0002177 | hsa-miR-486-5p | 1 | 0 | 0 |
| MIMAT0002808 | hsa-miR-511-5p | 1 | 0 | 0 |
| MIMAT0002810 | hsa-miR-202-5p | 1 | 0 | 0 |
| MIMAT0002811 | hsa-miR-202-3p | 1 | 0 | 0 |
| MIMAT0002816 | hsa-miR-494-3p | 1 | 0 | 0 |
| MIMAT0002819 | hsa-miR-193b-3p | 1 | 0 | 0 |
| MIMAT0002820 | hsa-miR-497-5p | 1 | 0 | 0 |
| MIMAT0002824 | hsa-miR-498 | 1 | 0 | 0 |
| MIMAT0002846 | hsa-miR-520c-3p | 1 | 0 | 0 |
| MIMAT0002875 | hsa-miR-504-5p | 1 | 0 | 0 |
| MIMAT0002879 | hsa-miR-507 | 1 | 0 | 0 |
| MIMAT0002891 | hsa-miR-18a-3p | 1 | 0 | 0 |
| MIMAT0003164 | hsa-miR-544a | 1 | 0 | 0 |
| MIMAT0003215 | hsa-miR-552-3p | 1 | 0 | 0 |
| MIMAT0003238 | hsa-miR-573 | 1 | 0 | 0 |
| MIMAT0003241 | hsa-miR-576-5p | 1 | 0 | 0 |
| MIMAT0003247 | hsa-miR-582-5p | 1 | 0 | 0 |
| MIMAT0003257 | hsa-miR-550a-3p | 1 | 0 | 0 |
| MIMAT0003258 | hsa-miR-590-5p | 1 | 0 | 0 |
| MIMAT0003293 | hsa-miR-624-5p | 1 | 0 | 0 |
| MIMAT0003311 | hsa-miR-641 | 1 | 0 | 0 |
| MIMAT0003322 | hsa-miR-652-3p | 1 | 0 | 0 |
| MIMAT0003327 | hsa-miR-449b-5p | 1 | 0 | 0 |
| MIMAT0003338 | hsa-miR-660-5p | 1 | 0 | 0 |
| MIMAT0003339 | hsa-miR-421 | 1 | 0 | 0 |
| MIMAT0003386 | hsa-miR-376a-5p | 1 | 0 | 0 |
| MIMAT0003389 | hsa-miR-542-3p | 1 | 0 | 0 |
| MIMAT0003887 | hsa-miR-769-3p | 1 | 0 | 0 |
| MIMAT0003945 | hsa-miR-765 | 1 | 0 | 0 |
| MIMAT0004484 | hsa-let-7d-3p | 1 | 0 | 0 |
| MIMAT0004493 | hsa-miR-20a-3p | 1 | 0 | 0 |
| MIMAT0004496 | hsa-miR-23a-5p | 1 | 0 | 0 |
| MIMAT0004497 | hsa-miR-24-2-5p | 1 | 0 | 0 |
| MIMAT0004498 | hsa-miR-25-5p | 1 | 0 | 0 |
| MIMAT0004500 | hsa-miR-26b-3p | 1 | 0 | 0 |
| MIMAT0004502 | hsa-miR-28-3p | 1 | 0 | 0 |
| MIMAT0004503 | hsa-miR-29a-5p | 1 | 0 | 0 |
| MIMAT0004507 | hsa-miR-92a-1-5p | 1 | 0 | 0 |
| MIMAT0004508 | hsa-miR-92a-2-5p | 1 | 0 | 0 |
| MIMAT0004515 | hsa-miR-29b-2-5p | 1 | 0 | 0 |
| MIMAT0004549 | hsa-miR-148a-5p | 1 | 0 | 0 |
| MIMAT0004551 | hsa-miR-30d-3p | 1 | 0 | 0 |
| MIMAT0004557 | hsa-miR-34a-3p | 1 | 0 | 0 |
| MIMAT0004559 | hsa-miR-181c-3p | 1 | 0 | 0 |
| MIMAT0004560 | hsa-miR-183-3p | 1 | 0 | 0 |
| MIMAT0004564 | hsa-miR-214-5p | 1 | 0 | 0 |
| MIMAT0004567 | hsa-miR-219a-1-3p | 1 | 0 | 0 |
| MIMAT0004569 | hsa-miR-222-5p | 1 | 0 | 0 |
| MIMAT0004584 | hsa-let-7g-3p | 1 | 0 | 0 |
| MIMAT0004592 | hsa-miR-125b-1-3p | 1 | 0 | 0 |
| MIMAT0004600 | hsa-miR-144-5p | 1 | 0 | 0 |
| MIMAT0004609 | hsa-miR-149-3p | 1 | 0 | 0 |
| MIMAT0004610 | hsa-miR-150-3p | 1 | 0 | 0 |
| MIMAT0004614 | hsa-miR-193a-5p | 1 | 0 | 0 |
| MIMAT0004673 | hsa-miR-29c-5p | 1 | 0 | 0 |
| MIMAT0004674 | hsa-miR-30c-1-3p | 1 | 0 | 0 |
| MIMAT0004678 | hsa-miR-99b-3p | 1 | 0 | 0 |
| MIMAT0004693 | hsa-miR-330-5p | 1 | 0 | 0 |
| MIMAT0004694 | hsa-miR-342-5p | 1 | 0 | 0 |
| MIMAT0004698 | hsa-miR-135b-3p | 1 | 0 | 0 |
| MIMAT0004703 | hsa-miR-335-3p | 1 | 0 | 0 |
| MIMAT0004749 | hsa-miR-424-3p | 1 | 0 | 0 |
| MIMAT0004764 | hsa-miR-490-5p | 1 | 0 | 0 |
| MIMAT0004768 | hsa-miR-497-3p | 1 | 0 | 0 |
| MIMAT0004770 | hsa-miR-516a-5p | 1 | 0 | 0 |
| MIMAT0004775 | hsa-miR-502-3p | 1 | 0 | 0 |
| MIMAT0004778 | hsa-miR-508-5p | 1 | 0 | 0 |
| MIMAT0004795 | hsa-miR-574-5p | 1 | 0 | 0 |
| MIMAT0004796 | hsa-miR-576-3p | 1 | 0 | 0 |
| MIMAT0004800 | hsa-miR-550a-5p | 1 | 0 | 0 |
| MIMAT0004805 | hsa-miR-616-3p | 1 | 0 | 0 |
| MIMAT0004806 | hsa-miR-548c-5p | 1 | 0 | 0 |
| MIMAT0004807 | hsa-miR-624-3p | 1 | 0 | 0 |
| MIMAT0004810 | hsa-miR-629-5p | 1 | 0 | 0 |
| MIMAT0004812 | hsa-miR-548d-5p | 1 | 0 | 0 |
| MIMAT0004814 | hsa-miR-654-3p | 1 | 0 | 0 |
| MIMAT0004819 | hsa-miR-671-3p | 1 | 0 | 0 |
| MIMAT0004911 | hsa-miR-874-3p | 1 | 0 | 0 |
| MIMAT0004925 | hsa-miR-876-3p | 1 | 0 | 0 |
| MIMAT0004926 | hsa-miR-708-5p | 1 | 0 | 0 |
| MIMAT0004946 | hsa-miR-744-3p | 1 | 0 | 0 |
| MIMAT0004947 | hsa-miR-885-5p | 1 | 0 | 0 |
| MIMAT0004949 | hsa-miR-877-5p | 1 | 0 | 0 |
| MIMAT0004950 | hsa-miR-877-3p | 1 | 0 | 0 |
| MIMAT0004953 | hsa-miR-873-5p | 1 | 0 | 0 |
| MIMAT0004955 | hsa-miR-374b-5p | 1 | 0 | 0 |
| MIMAT0004957 | hsa-miR-760 | 1 | 0 | 0 |
| MIMAT0004958 | hsa-miR-301b-3p | 1 | 0 | 0 |
| MIMAT0004978 | hsa-miR-935 | 1 | 0 | 0 |
| MIMAT0005451 | hsa-miR-522-5p | 1 | 0 | 0 |
| MIMAT0005458 | hsa-miR-1224-5p | 1 | 0 | 0 |
| MIMAT0005572 | hsa-miR-1225-5p | 1 | 0 | 0 |
| MIMAT0005576 | hsa-miR-1226-5p | 1 | 0 | 0 |
| MIMAT0005577 | hsa-miR-1226-3p | 1 | 0 | 0 |
| MIMAT0005584 | hsa-miR-1229-3p | 1 | 0 | 0 |
| MIMAT0005586 | hsa-miR-1231 | 1 | 0 | 0 |
| MIMAT0005825 | hsa-miR-1180-3p | 1 | 0 | 0 |
| MIMAT0005865 | hsa-miR-1202 | 1 | 0 | 0 |
| MIMAT0005883 | hsa-miR-1293 | 1 | 0 | 0 |
| MIMAT0005901 | hsa-miR-1249-3p | 1 | 0 | 0 |
| MIMAT0005905 | hsa-miR-1254 | 1 | 0 | 0 |
| MIMAT0005915 | hsa-miR-1263 | 1 | 0 | 0 |
| MIMAT0005942 | hsa-miR-1288-3p | 1 | 0 | 0 |
| MIMAT0005948 | hsa-miR-664a-5p | 1 | 0 | 0 |
| MIMAT0006789 | hsa-miR-1468-5p | 1 | 0 | 0 |
| MIMAT0007402 | hsa-miR-103b | 1 | 0 | 0 |
| MIMAT0007884 | hsa-miR-1910-5p | 1 | 0 | 0 |
| MIMAT0009196 | hsa-miR-103a-2-5p | 1 | 0 | 0 |
| MIMAT0009451 | hsa-miR-1976 | 1 | 0 | 0 |
| MIMAT0010195 | hsa-let-7a-2-3p | 1 | 0 | 0 |
| MIMAT0010214 | hsa-miR-151b | 1 | 0 | 0 |
| MIMAT0010251 | hsa-miR-449c-5p | 1 | 0 | 0 |
| MIMAT0011777 | hsa-miR-2277-3p | 1 | 0 | 0 |
| MIMAT0011778 | hsa-miR-2278 | 1 | 0 | 0 |
| MIMAT0015008 | hsa-miR-3140-3p | 1 | 0 | 0 |
| MIMAT0015032 | hsa-miR-3158-3p | 1 | 0 | 0 |
| MIMAT0015069 | hsa-miR-3187-3p | 1 | 0 | 0 |
| MIMAT0016888 | hsa-miR-4326 | 1 | 0 | 0 |
| MIMAT0016895 | hsa-miR-2355-5p | 1 | 0 | 0 |
| MIMAT0017986 | hsa-miR-3609 | 1 | 0 | 0 |
| MIMAT0017990 | hsa-miR-3613-5p | 1 | 0 | 0 |
| MIMAT0017994 | hsa-miR-3615 | 1 | 0 | 0 |
| MIMAT0018073 | hsa-miR-3653-3p | 1 | 0 | 0 |
| MIMAT0018104 | hsa-miR-3679-5p | 1 | 0 | 0 |
| MIMAT0018349 | hsa-miR-3934-5p | 1 | 0 | 0 |
| MIMAT0018356 | hsa-miR-3940-3p | 1 | 0 | 0 |
| MIMAT0018443 | hsa-miR-374c-5p | 1 | 0 | 0 |
| MIMAT0018926 | hsa-miR-378d | 1 | 0 | 0 |
| MIMAT0018934 | hsa-miR-4421 | 1 | 0 | 0 |
| MIMAT0018940 | hsa-miR-4425 | 1 | 0 | 0 |
| MIMAT0019061 | hsa-miR-4523 | 1 | 0 | 0 |
| MIMAT0019197 | hsa-miR-3117-5p | 1 | 0 | 0 |
| MIMAT0019201 | hsa-miR-3127-3p | 1 | 0 | 0 |
| MIMAT0019208 | hsa-miR-3074-5p | 1 | 0 | 0 |
| MIMAT0019731 | hsa-miR-4662a-5p | 1 | 0 | 0 |
| MIMAT0019737 | hsa-miR-4664-5p | 1 | 0 | 0 |
| MIMAT0019738 | hsa-miR-4664-3p | 1 | 0 | 0 |
| MIMAT0019772 | hsa-miR-4685-3p | 1 | 0 | 0 |
| MIMAT0019952 | hsa-miR-2467-5p | 1 | 0 | 0 |
| MIMAT0021020 | hsa-miR-5000-3p | 1 | 0 | 0 |
| MIMAT0021044 | hsa-miR-5010-3p | 1 | 0 | 0 |
| MIMAT0022259 | hsa-miR-5100 | 1 | 0 | 0 |
| MIMAT0022691 | hsa-miR-197-5p | 1 | 0 | 0 |
| MIMAT0022692 | hsa-miR-181b-3p | 1 | 0 | 0 |
| MIMAT0022720 | hsa-miR-1304-3p | 1 | 0 | 0 |
| MIMAT0022741 | hsa-miR-3529-3p | 1 | 0 | 0 |
| MIMAT0022844 | hsa-miR-216a-3p | 1 | 0 | 0 |
| MIMAT0025479 | hsa-miR-6511a-3p | 1 | 0 | 0 |
| MIMAT0026477 | hsa-miR-128-1-5p | 1 | 0 | 0 |
| MIMAT0030021 | hsa-miR-7706 | 1 | 0 | 0 |
| MIMAT0030415 | hsa-miR-1273h-5p | 1 | 0 | 0 |
| MIMAT0030426 | hsa-miR-7851-3p | 1 | 0 | 0 |
| MIMAT0037327 | hsa-miR-519a-2-5p | 1 | 0 | 0 |
| MIMAT0037325 | hsa-miR-520b-5p | 1 | 0 | 0 |
| MIMAT0039323 | hsa-miR-9903 | 1 | 0 | 0 |

Supplementary Table 3: Details of the TFs-genes regulatory network.

| **Id** | **Label** | **Degree** | **Betweenness** | **Expression** |
| --- | --- | --- | --- | --- |
| 2023 | ENO1 | 14 | 274.57 | 0 |
| 2171 | FABP5 | 12 | 246.59 | 0 |
| 4288 | MKI67 | 7 | 130.01 | 0 |
| 10200 | MPHOSPH6 | 6 | 97.84 | 0 |
| 2296 | FOXC1 | 3 | 79.89 | 0 |
| 4782 | NFIC | 3 | 79.89 | 0 |
| 25988 | HINFP | 2 | 58.95 | 0 |
| 7392 | USF2 | 2 | 41.18 | 0 |
| 7022 | TFAP2C | 2 | 26.9 | 0 |
| 4205 | MEF2A | 2 | 25.19 | 0 |
| 5970 | RELA | 2 | 25.19 | 0 |
| 4800 | NFYA | 2 | 11.81 | 0 |
| 1820 | ARID3A | 1 | 0 | 0 |
| 3725 | JUN | 1 | 0 | 0 |
| 1385 | CREB1 | 1 | 0 | 0 |
| 2624 | GATA2 | 1 | 0 | 0 |
| 2300 | FOXL1 | 1 | 0 | 0 |
| 7528 | YY1 | 1 | 0 | 0 |
| 3202 | HOXA5 | 1 | 0 | 0 |
| 6722 | SRF | 1 | 0 | 0 |
| 6774 | STAT3 | 1 | 0 | 0 |
| 1869 | E2F1 | 1 | 0 | 0 |
| 1876 | E2F6 | 1 | 0 | 0 |
| 688 | KLF5 | 1 | 0 | 0 |
| 4149 | MAX | 1 | 0 | 0 |
| 7391 | USF1 | 1 | 0 | 0 |
| 6772 | STAT1 | 1 | 0 | 0 |
| 3660 | IRF2 | 1 | 0 | 0 |
| 639 | PRDM1 | 1 | 0 | 0 |
| 8626 | TP63 | 1 | 0 | 0 |
| 860 | RUNX2 | 1 | 0 | 0 |
| 4899 | NRF1 | 1 | 0 | 0 |
| 2001 | ELF5 | 1 | 0 | 0 |

Supplementary Table 4: Drugs predicted to target biomarkers in the DSigDB database.

| **Term** | **Overlap** | **P-value** | **Adjusted P-value** | **Old P-value** | **Old Adjusted P-value** | **Odds Ratio** | **Combined Score** | **Genes** |
| --- | --- | --- | --- | --- | --- | --- | --- | --- |
| rosiglitazone CTD 00003139 | 3/411 | 3.3936336997534054E-5 | 0.0073422088777743725 | 0 | 0 | 144.02941176470588 | 1482.210166275378 | FABP5;ENO1;MKI67 |
| 3-Butylidenephthalide CTD 00001227 | 2/58 | 4.94051825135443E-5 | 0.0073422088777743725 | 0 | 0 | 356.07142857142856 | 3530.6103087148736 | ENO1;MKI67 |
| curcumin CTD 00000663 | 3/528 | 7.174796948965185E-5 | 0.0073422088777743725 | 0 | 0 | 111.26285714285714 | 1061.7092365117326 | ENO1;MKI67;MPHOSPH6 |
| Dibenzo[def,p]chrysene CTD 00001899 | 2/84 | 1.0401011460061032E-4 | 0.007982776295596843 | 0 | 0 | 242.85365853658536 | 2227.2163442496094 | ENO1;MKI67 |
| clomipramine PC3 DOWN | 2/108 | 1.7211975151281762E-4 | 0.010568152742887001 | 0 | 0 | 187.64150943396226 | 1626.3490251255419 | ENO1;MKI67 |
| 7646-79-9 CTD 00000928 | 4/3094 | 5.717912265594468E-4 | 0.02925665109229169 | 0 | 0 | 67624.0 | 504930.597308643 | FABP5;ENO1;MKI67;MPHOSPH6 |
| Retinoic acid BOSS | 2/232 | 7.916255621933881E-4 | 0.03471843537048145 | 0 | 0 | 85.93913043478261 | 613.7276012984353 | FABP5;ENO1 |
| Zinc sulfate CTD 00007264 | 2/287 | 0.0012079927480026823 | 0.04308033453256256 | 0 | 0 | 69.16140350877193 | 464.68130473018675 | FABP5;MPHOSPH6 |
| MS-275 PC3 DOWN | 2/294 | 0.0012671471920907573 | 0.04308033453256256 | 0 | 0 | 67.47945205479452 | 450.154561641271 | ENO1;MKI67 |
| acetaminophen CTD 00005295 | 4/4135 | 0.0018250458613839513 | 0.04308033453256256 | 0 | 0 | 63460.0 | 400188.2893273516 | FABP5;ENO1;MKI67;MPHOSPH6 |
| Paraoxon-methyl CTD 00000411 | 1/15 | 0.0029968027587318545 | 0.04308033453256256 | 0 | 0 | 475.76190476190476 | 2764.2762463526265 | MKI67 |
| ABT-737 CTD 00004466 | 1/18 | 0.003595357627375994 | 0.04308033453256256 | 0 | 0 | 391.7450980392157 | 2204.7852142154165 | MKI67 |
| ketamine CTD 00006188 | 1/19 | 0.0037948161126764622 | 0.04308033453256256 | 0 | 0 | 369.962962962963 | 2062.2177011886433 | MKI67 |
| TRIAMCINOLONE ACETONIDE CTD 00006922 | 1/20 | 0.003994244696037095 | 0.04308033453256256 | 0 | 0 | 350.4736842105263 | 1935.6313838807391 | MKI67 |
| aminoguanidine CTD 00000144 | 1/20 | 0.003994244696037095 | 0.04308033453256256 | 0 | 0 | 350.4736842105263 | 1935.6313838807391 | MKI67 |
| primaquine PC3 DOWN | 2/535 | 0.0041347260901326306 | 0.04308033453256256 | 0 | 0 | 36.51594746716698 | 200.41172312885413 | ENO1;MKI67 |
| salsolidin PC3 UP | 1/22 | 0.004393012155412379 | 0.04308033453256256 | 0 | 0 | 317.06349206349205 | 1720.9382450199414 | ENO1 |
| naftopidil HL60 DOWN | 1/22 | 0.004393012155412379 | 0.04308033453256256 | 0 | 0 | 317.06349206349205 | 1720.9382450199414 | ENO1 |
| sisomicin HL60 DOWN | 1/23 | 0.004592351033521324 | 0.04308033453256256 | 0 | 0 | 302.6363636363636 | 1629.2014564159526 | ENO1 |
| tolbutamide MCF7 DOWN | 1/23 | 0.004592351033521324 | 0.04308033453256256 | 0 | 0 | 302.6363636363636 | 1629.2014564159526 | ENO1 |
| METHYL PARATHION CTD 00006309 | 1/23 | 0.004592351033521324 | 0.04308033453256256 | 0 | 0 | 302.6363636363636 | 1629.2014564159526 | MKI67 |
| NICKEL SULFATE CTD 00001417 | 2/576 | 0.00478008604069279 | 0.04308033453256256 | 0 | 0 | 33.836236933797906 | 180.79705424831735 | FABP5;MKI67 |
| dipivefrine HL60 DOWN | 1/24 | 0.004791660011821214 | 0.04308033453256256 | 0 | 0 | 289.463768115942 | 1545.9907779727164 | ENO1 |
| estropipate PC3 UP | 1/24 | 0.004791660011821214 | 0.04308033453256256 | 0 | 0 | 289.463768115942 | 1545.9907779727164 | ENO1 |
| ifosfamide MCF7 UP | 1/24 | 0.004791660011821214 | 0.04308033453256256 | 0 | 0 | 289.463768115942 | 1545.9907779727164 | ENO1 |
| 7-aminocephalosporanic acid PC3 UP | 1/24 | 0.004791660011821214 | 0.04308033453256256 | 0 | 0 | 289.463768115942 | 1545.9907779727164 | ENO1 |
| SC-560 MCF7 DOWN | 1/24 | 0.004791660011821214 | 0.04308033453256256 | 0 | 0 | 289.463768115942 | 1545.9907779727164 | ENO1 |
| diphenylpyraline PC3 DOWN | 1/25 | 0.00499093909080392 | 0.04308033453256256 | 0 | 0 | 277.3888888888889 | 1470.1975024182984 | ENO1 |
| letrozole CTD 00002692 | 1/25 | 0.00499093909080392 | 0.04308033453256256 | 0 | 0 | 277.3888888888889 | 1470.1975024182984 | MKI67 |
| benfluorex MCF7 DOWN | 1/26 | 0.005190188272057036 | 0.04308033453256256 | 0 | 0 | 266.28 | 1400.8951674244283 | ENO1 |
| amprolium PC3 UP | 1/26 | 0.005190188272057036 | 0.04308033453256256 | 0 | 0 | 266.28 | 1400.8951674244283 | ENO1 |
| propantheline bromide PC3 DOWN | 1/27 | 0.005389407557575397 | 0.04308033453256256 | 0 | 0 | 256.02564102564105 | 1337.303803970803 | ENO1 |
| CI-1040 CTD 00003697 | 1/27 | 0.005389407557575397 | 0.04308033453256256 | 0 | 0 | 256.02564102564105 | 1337.303803970803 | MKI67 |
| isoxsuprine HL60 DOWN | 1/27 | 0.005389407557575397 | 0.04308033453256256 | 0 | 0 | 256.02564102564105 | 1337.303803970803 | ENO1 |
| nicotinic acid PC3 DOWN | 1/27 | 0.005389407557575397 | 0.04308033453256256 | 0 | 0 | 256.02564102564105 | 1337.303803970803 | ENO1 |
| ambroxol PC3 DOWN | 2/622 | 0.005557406519513491 | 0.04308033453256256 | 0 | 0 | 31.251612903225805 | 162.27786685168638 | ENO1;MKI67 |
| quercetin PC3 DOWN | 1/28 | 0.005588596948698618 | 0.04308033453256256 | 0 | 0 | 246.53086419753086 | 1278.7622529681282 | ENO1 |
| 0316684-0000 MCF7 DOWN | 1/28 | 0.005588596948698618 | 0.04308033453256256 | 0 | 0 | 246.53086419753086 | 1278.7622529681282 | ENO1 |
| 7-Hydroxystaurosporine CTD 00002331 | 1/30 | 0.0059868860535258585 | 0.04308033453256256 | 0 | 0 | 229.50574712643677 | 1174.6526106132212 | MKI67 |
| succinylsulfathiazole PC3 DOWN | 1/30 | 0.0059868860535258585 | 0.04308033453256256 | 0 | 0 | 229.50574712643677 | 1174.6526106132212 | ENO1 |
| meglumine PC3 DOWN | 1/31 | 0.0061859857715872765 | 0.04308033453256256 | 0 | 0 | 221.84444444444443 | 1128.183023929502 | ENO1 |
| Gly-His-Lys PC3 UP | 1/31 | 0.0061859857715872765 | 0.04308033453256256 | 0 | 0 | 221.84444444444443 | 1128.183023929502 | ENO1 |
| Cobalt sulfate CTD 00001238 | 1/32 | 0.006385055602597088 | 0.04308033453256256 | 0 | 0 | 214.67741935483872 | 1084.93568598441 | ENO1 |
| simvastatin PC3 UP | 1/32 | 0.006385055602597088 | 0.04308033453256256 | 0 | 0 | 214.67741935483872 | 1084.93568598441 | ENO1 |
| alexidine PC3 DOWN | 1/34 | 0.0067831056106472404 | 0.04308033453256256 | 0 | 0 | 201.64646464646464 | 1006.8853706700453 | MKI67 |
| scriptaid PC3 DOWN | 2/692 | 0.0068471282593910165 | 0.04308033453256256 | 0 | 0 | 27.979710144927537 | 139.44880337569757 | ENO1;MKI67 |
| hyoscyamine HL60 UP | 1/36 | 0.00718103609555633 | 0.04308033453256256 | 0 | 0 | 190.10476190476192 | 938.4163420388405 | ENO1 |
| Selenium methyl cysteine CTD 00000103 | 1/37 | 0.007379956522320185 | 0.04308033453256256 | 0 | 0 | 184.8148148148148 | 907.2536215941285 | MKI67 |
| EXEMESTANE CTD 00002383 | 1/37 | 0.007379956522320185 | 0.04308033453256256 | 0 | 0 | 184.8148148148148 | 907.2536215941285 | MKI67 |
| puromycin PC3 DOWN | 2/722 | 0.007438923424167953 | 0.04308033453256256 | 0 | 0 | 26.772222222222222 | 131.21144129810958 | ENO1;MKI67 |
| Ephedrone CTD 00002112 | 1/38 | 0.00757884707428762 | 0.04308033453256256 | 0 | 0 | 179.8108108108108 | 877.9072583460236 | FABP5 |
| pyrantel HL60 UP | 1/39 | 0.007777707754316145 | 0.04308033453256256 | 0 | 0 | 175.0701754385965 | 850.2271896016904 | ENO1 |
| amphotericin b CTD 00005393 | 1/39 | 0.007777707754316145 | 0.04308033453256256 | 0 | 0 | 175.0701754385965 | 850.2271896016904 | ENO1 |
| arsenite BOSS | 1/41 | 0.008175339510378254 | 0.04308033453256256 | 0 | 0 | 166.3 | 799.343073344609 | ENO1 |
| ACRYLAMIDE CTD 00007343 | 1/42 | 0.008374110589681004 | 0.04308033453256256 | 0 | 0 | 162.2357723577236 | 775.9104929668098 | MKI67 |
| phosphoenolpyruvate BOSS | 1/42 | 0.008374110589681004 | 0.04308033453256256 | 0 | 0 | 162.2357723577236 | 775.9104929668098 | ENO1 |
| amantadine PC3 DOWN | 1/42 | 0.008374110589681004 | 0.04308033453256256 | 0 | 0 | 162.2357723577236 | 775.9104929668098 | ENO1 |
| 0173570-0000 PC3 DOWN | 1/43 | 0.008572851807857029 | 0.04308033453256256 | 0 | 0 | 158.36507936507937 | 753.6839332107746 | MKI67 |
| hydrogen peroxide CTD 00006118 | 3/2673 | 0.008584486164140408 | 0.04308033453256256 | 0 | 0 | 19.467415730337077 | 92.62204407003311 | FABP5;ENO1;MPHOSPH6 |
| pentetrazol HL60 UP | 1/44 | 0.008771563165193935 | 0.04308033453256256 | 0 | 0 | 154.67441860465115 | 732.5752067987863 | ENO1 |
| Toluidine Blue O BOSS | 1/45 | 0.008970244665779415 | 0.04308033453256256 | 0 | 0 | 151.15151515151516 | 712.5044099532315 | ENO1 |
| colchicine SKMEL5 UP | 1/46 | 0.009168896311972097 | 0.04308033453256256 | 0 | 0 | 147.78518518518518 | 693.3989791915002 | ENO1 |
| benserazide PC3 UP | 1/47 | 0.009367518105959016 | 0.04308033453256256 | 0 | 0 | 144.56521739130434 | 675.1928734367539 | ENO1 |
| primidone PC3 UP | 1/48 | 0.009566110051223143 | 0.04308033453256256 | 0 | 0 | 141.4822695035461 | 657.8258626058753 | ENO1 |
| Phytoestrogens CTD 00007437 | 1/48 | 0.009566110051223143 | 0.04308033453256256 | 0 | 0 | 141.4822695035461 | 657.8258626058753 | MKI67 |
| etoposide MCF7 DOWN | 1/48 | 0.009566110051223143 | 0.04308033453256256 | 0 | 0 | 141.4822695035461 | 657.8258626058753 | MKI67 |
| ronidazole MCF7 DOWN | 1/49 | 0.009764672150221917 | 0.04308033453256256 | 0 | 0 | 138.52777777777777 | 641.2429069487843 | ENO1 |
| domperidone MCF7 DOWN | 1/49 | 0.009764672150221917 | 0.04308033453256256 | 0 | 0 | 138.52777777777777 | 641.2429069487843 | ENO1 |
| EMBELIN CTD 00000580 | 1/49 | 0.009764672150221917 | 0.04308033453256256 | 0 | 0 | 138.52777777777777 | 641.2429069487843 | MKI67 |
| disopyramide MCF7 DOWN | 1/50 | 0.009963204403296228 | 0.04308033453256256 | 0 | 0 | 135.69387755102042 | 625.3936138885244 | ENO1 |
| CP-863187 PC3 DOWN | 1/50 | 0.009963204403296228 | 0.04308033453256256 | 0 | 0 | 135.69387755102042 | 625.3936138885244 | ENO1 |
| methotrexate MCF7 DOWN | 1/52 | 0.010360179389191183 | 0.04321995990355682 | 0 | 0 | 130.359477124183 | 595.714877907605 | MKI67 |
| thioridazine PC3 DOWN | 1/53 | 0.010558622126276096 | 0.04321995990355682 | 0 | 0 | 127.84615384615384 | 581.8038736810901 | ENO1 |
| LY-294002 HL60 DOWN | 1/53 | 0.010558622126276096 | 0.04321995990355682 | 0 | 0 | 127.84615384615384 | 581.8038736810901 | FABP5 |
| alprostadil PC3 UP | 1/53 | 0.010558622126276096 | 0.04321995990355682 | 0 | 0 | 127.84615384615384 | 581.8038736810901 | ENO1 |
| cadmium acetate CTD 00001325 | 1/54 | 0.010757035030210377 | 0.043344402586283255 | 0 | 0 | 125.42767295597484 | 568.4627119975241 | ENO1 |
| zidovudine PC3 UP | 1/55 | 0.010955418103578725 | 0.043344402586283255 | 0 | 0 | 123.09876543209876 | 555.6581197291017 | ENO1 |
| 5707885 MCF7 UP | 1/56 | 0.011153771349564747 | 0.043344402586283255 | 0 | 0 | 120.85454545454546 | 543.3593292987222 | ENO1 |
| nilutamide PC3 UP | 1/56 | 0.011153771349564747 | 0.043344402586283255 | 0 | 0 | 120.85454545454546 | 543.3593292987222 | ENO1 |
| quinisocaine PC3 UP | 1/58 | 0.011550388364883564 | 0.04370601131397418 | 0 | 0 | 116.60233918128655 | 520.1672581812571 | ENO1 |
| H-89 PC3 DOWN | 1/60 | 0.011946886102556297 | 0.04370601131397418 | 0 | 0 | 112.63841807909604 | 498.6823350537484 | ENO1 |
| STOCK1N-35696 PC3 DOWN | 1/60 | 0.011946886102556297 | 0.04370601131397418 | 0 | 0 | 112.63841807909604 | 498.6823350537484 | ENO1 |
| ciclopirox HL60 DOWN | 1/61 | 0.012145090247431321 | 0.04370601131397418 | 0 | 0 | 110.75555555555556 | 488.52395875680094 | MKI67 |
| cefotiam PC3 UP | 1/62 | 0.012343264582388234 | 0.04370601131397418 | 0 | 0 | 108.93442622950819 | 478.7281035203865 | ENO1 |
| Iniprol BOSS | 1/62 | 0.012343264582388234 | 0.04370601131397418 | 0 | 0 | 108.93442622950819 | 478.7281035203865 | ENO1 |
| orciprenaline PC3 UP | 1/63 | 0.012541409107047853 | 0.04370601131397418 | 0 | 0 | 107.17204301075269 | 469.27630184403733 | ENO1 |
| 67526-95-8 CTD 00007263 | 2/953 | 0.012761837870854846 | 0.04370601131397418 | 0 | 0 | 20.02628811777077 | 87.34056981572033 | MKI67;MPHOSPH6 |
| Melatonin CTD 00006260 | 1/66 | 0.013135663858227446 | 0.04370601131397418 | 0 | 0 | 102.2102564102564 | 442.81820021177793 | MKI67 |
| BAS-012416453 PC3 UP | 1/66 | 0.013135663858227446 | 0.04370601131397418 | 0 | 0 | 102.2102564102564 | 442.81820021177793 | ENO1 |
| chlortetracycline PC3 UP | 1/67 | 0.013333689173978527 | 0.04370601131397418 | 0 | 0 | 100.65656565656566 | 434.58083948016025 | ENO1 |
| ciclopirox MCF7 DOWN | 1/67 | 0.013333689173978527 | 0.04370601131397418 | 0 | 0 | 100.65656565656566 | 434.58083948016025 | MKI67 |
| creatine BOSS | 1/67 | 0.013333689173978527 | 0.04370601131397418 | 0 | 0 | 100.65656565656566 | 434.58083948016025 | ENO1 |
| 5109870 MCF7 DOWN | 1/68 | 0.013531684697143435 | 0.04370601131397418 | 0 | 0 | 99.14925373134328 | 426.61160877153185 | MKI67 |
| OXYTOCIN BOSS | 1/69 | 0.013729650427898048 | 0.04370601131397418 | 0 | 0 | 97.68627450980392 | 418.8980400895514 | ENO1 |
| spiperone PC3 DOWN | 1/69 | 0.013729650427898048 | 0.04370601131397418 | 0 | 0 | 97.68627450980392 | 418.8980400895514 | MKI67 |
| tonzonium bromide PC3 DOWN | 1/70 | 0.013927586368181954 | 0.04370601131397418 | 0 | 0 | 96.26570048309179 | 411.4284153662677 | MKI67 |
| 136572-09-3 BOSS | 1/70 | 0.013927586368181954 | 0.04370601131397418 | 0 | 0 | 96.26570048309179 | 411.4284153662677 | ENO1 |
| nitrofural PC3 UP | 1/71 | 0.014125492523803207 | 0.04370601131397418 | 0 | 0 | 94.88571428571429 | 404.19171136393726 | ENO1 |
| PARAOXON CTD 00006470 | 1/72 | 0.01432336889462672 | 0.04370601131397418 | 0 | 0 | 93.54460093896714 | 397.1775494166316 | ENO1 |
| Benzo[b]fluoranthene CTD 00000306 | 1/73 | 0.014521215485424647 | 0.04370601131397418 | 0 | 0 | 92.24074074074075 | 390.3761493181644 | MKI67 |
| benzocaine PC3 DOWN | 1/73 | 0.014521215485424647 | 0.04370601131397418 | 0 | 0 | 92.24074074074075 | 390.3761493181644 | ENO1 |
| PHA-00767505E MCF7 DOWN | 1/73 | 0.014521215485424647 | 0.04370601131397418 | 0 | 0 | 92.24074074074075 | 390.3761493181644 | ENO1 |
| chlortetracycline HL60 DOWN | 2/1050 | 0.015390610252357778 | 0.04587298395605668 | 0 | 0 | 18.080152671755727 | 75.46651529497264 | FABP5;MPHOSPH6 |
| microcystin RR CTD 00002594 | 1/88 | 0.01748534258913372 | 0.05147079238916285 | 0 | 0 | 76.27969348659003 | 308.6575655408228 | FABP5 |
| trichostatin A PC3 DOWN | 2/1133 | 0.017819103656822616 | 0.05147079238916285 | 0 | 0 | 16.679929266136163 | 67.17815087669386 | ENO1;MKI67 |
| (+)-chelidonine HL60 UP | 1/90 | 0.017880053832220178 | 0.05147079238916285 | 0 | 0 | 74.55805243445693 | 300.0267846683708 | MPHOSPH6 |
| 7,8-Benzoflavone CTD 00000606 | 1/91 | 0.018077364860211664 | 0.05147079238916285 | 0 | 0 | 73.72592592592592 | 295.8691213711147 | ENO1 |
| pyrvinium MCF7 DOWN | 1/92 | 0.018274646157715797 | 0.05147079238916285 | 0 | 0 | 72.91208791208791 | 291.8117210702858 | MKI67 |
| Diallyl trisulfide CTD 00001934 | 1/92 | 0.018274646157715797 | 0.05147079238916285 | 0 | 0 | 72.91208791208791 | 291.8117210702858 | ENO1 |
| Deguelin CTD 00003487 | 1/93 | 0.018471897734518974 | 0.051553387313612045 | 0 | 0 | 72.1159420289855 | 287.8511246681871 | MKI67 |
| Cornstarch BOSS | 1/95 | 0.018866311727459114 | 0.05217979910207161 | 0 | 0 | 70.57446808510639 | 280.20727279514847 | ENO1 |
| 0297417-0002B MCF7 DOWN | 1/99 | 0.019654783165825862 | 0.053423788919118115 | 0 | 0 | 67.68027210884354 | 265.94519972543833 | ENO1 |
| trichostatin A CTD 00000660 | 3/3584 | 0.019912946300987992 | 0.053423788919118115 | 0 | 0 | 13.751745322535605 | 53.85713173007475 | FABP5;ENO1;MKI67 |
| amantadine HL60 DOWN | 1/102 | 0.020245824851614903 | 0.053423788919118115 | 0 | 0 | 65.66006600660066 | 256.06156447186925 | ENO1 |
| resveratrol MCF7 DOWN | 1/104 | 0.020639704165072065 | 0.053423788919118115 | 0 | 0 | 64.37864077669903 | 249.82380515639142 | MKI67 |
| AG-028671 MCF7 UP | 1/104 | 0.020639704165072065 | 0.053423788919118115 | 0 | 0 | 64.37864077669903 | 249.82380515639142 | ENO1 |
| Pinosylvin CTD 00002139 | 1/105 | 0.020836599290491847 | 0.053423788919118115 | 0 | 0 | 63.756410256410255 | 246.8038857860016 | ENO1 |
| MAGNESIUM BOSS | 1/105 | 0.020836599290491847 | 0.053423788919118115 | 0 | 0 | 63.756410256410255 | 246.8038857860016 | ENO1 |
| danazol HL60 DOWN | 2/1233 | 0.020959711956770863 | 0.053423788919118115 | 0 | 0 | 15.24370430544273 | 58.91925189897311 | FABP5;MKI67 |
| buflomedil PC3 DOWN | 1/106 | 0.02103346472728197 | 0.053423788919118115 | 0 | 0 | 63.146031746031746 | 243.84727683680757 | ENO1 |
| 1,10-phenanthroline CTD 00001181 | 1/107 | 0.021230300482515996 | 0.053423788919118115 | 0 | 0 | 62.54716981132076 | 240.952079089247 | ENO1 |
| (2E,4E,6E,8E)-3,7-DIMETHYL-9-(2,6,6-TRIMETHYL-3-OXO-1-CYCLOHEXENYL)NONA-2,4,6,8-TETRAENOIC ACID CTD 00000070 | 1/107 | 0.021230300482515996 | 0.053423788919118115 | 0 | 0 | 62.54716981132076 | 240.952079089247 | FABP5 |
| chlorpromazine CTD 00005648 | 1/113 | 0.022410691845982666 | 0.05579450234269561 | 0 | 0 | 59.17857142857143 | 224.7730631043994 | MKI67 |
| Arsenenous acid CTD 00000922 | 2/1283 | 0.022616419151618935 | 0.05579450234269561 | 0 | 0 | 14.60967993754879 | 55.35723327943678 | FABP5;ENO1 |
| chlorzoxazone HL60 DOWN | 2/1286 | 0.022717631247025902 | 0.05579450234269561 | 0 | 0 | 14.573208722741432 | 55.15396901462181 | MKI67;MPHOSPH6 |
| celecoxib CTD 00003448 | 1/116 | 0.023000487051863392 | 0.05604086924541318 | 0 | 0 | 57.62608695652174 | 217.37942375541598 | MKI67 |
| deferoxamine CTD 00005759 | 1/123 | 0.024375638130629797 | 0.05892378666223109 | 0 | 0 | 54.30054644808743 | 201.6815193935306 | ENO1 |
| METHYL METHANESULFONATE CTD 00006307 | 3/3864 | 0.024652955189909974 | 0.05894464661518004 | 0 | 0 | 12.536907536907536 | 46.4223946327251 | FABP5;ENO1;MKI67 |
| oxiglutatione BOSS | 1/125 | 0.024768271704749923 | 0.05894464661518004 | 0 | 0 | 53.41935483870968 | 197.5550206477932 | ENO1 |
| citric acid BOSS | 1/126 | 0.024964544047929384 | 0.05895473094395632 | 0 | 0 | 52.989333333333335 | 195.54646785283802 | ENO1 |
| methyprylon BOSS | 1/127 | 0.025160786767501147 | 0.05896459189025078 | 0 | 0 | 52.56613756613756 | 193.57314980526166 | ENO1 |
| paclitaxel PC3 UP | 1/129 | 0.02555318334636167 | 0.05943050975252297 | 0 | 0 | 51.739583333333336 | 189.7287094266052 | ENO1 |
| STOCK1N-28457 MCF7 DOWN | 1/132 | 0.02614155610795585 | 0.05946885659409423 | 0 | 0 | 50.5470737913486 | 184.20511433882353 | ENO1 |
| ACRYLAMIDE BOSS | 1/133 | 0.026337621148016778 | 0.05946885659409423 | 0 | 0 | 50.16161616161616 | 182.4256036925459 | ENO1 |
| Adenosine triphosphate BOSS | 1/134 | 0.0265336565820586 | 0.05946885659409423 | 0 | 0 | 49.78195488721804 | 180.67570447529343 | ENO1 |
| 9001-31-4 BOSS | 1/135 | 0.026729662413721805 | 0.05946885659409423 | 0 | 0 | 49.407960199004975 | 178.95471178559626 | ENO1 |
| AG-012559 PC3 DOWN | 1/135 | 0.026729662413721805 | 0.05946885659409423 | 0 | 0 | 49.407960199004975 | 178.95471178559626 | ENO1 |
| MeIQx CTD 00001739 | 1/136 | 0.02692563865335211 | 0.05946885659409423 | 0 | 0 | 49.03950617283951 | 177.26194251685567 | MKI67 |
| Ethene BOSS | 1/136 | 0.02692563865335211 | 0.05946885659409423 | 0 | 0 | 49.03950617283951 | 177.26194251685567 | ENO1 |
| thalidomide CTD 00006858 | 1/141 | 0.0279050759783193 | 0.061168962940175944 | 0 | 0 | 47.27619047619048 | 169.1989645694177 | MKI67 |
| CADMIUM BOSS | 1/143 | 0.028296643826728248 | 0.061168962940175944 | 0 | 0 | 46.605633802816904 | 166.14964722629514 | ENO1 |
| R-atenolol PC3 DOWN | 1/143 | 0.028296643826728248 | 0.061168962940175944 | 0 | 0 | 46.605633802816904 | 166.14964722629514 | ENO1 |
| cefadroxil HL60 DOWN | 1/144 | 0.028492383389072184 | 0.061168962940175944 | 0 | 0 | 46.277389277389275 | 164.66043385202047 | ENO1 |
| fulvestrant PC3 DOWN | 1/146 | 0.02888377380927212 | 0.06157860110726765 | 0 | 0 | 45.63448275862069 | 161.75029703137994 | ENO1 |
| ibuprofen CTD 00006137 | 1/149 | 0.029470637700984677 | 0.061955941261482975 | 0 | 0 | 44.7027027027027 | 157.54845500306016 | ENO1 |
| Zinc acetate dihydrate CTD 00007284 | 1/149 | 0.029470637700984677 | 0.061955941261482975 | 0 | 0 | 44.7027027027027 | 157.54845500306016 | ENO1 |
| thioguanosine MCF7 DOWN | 1/150 | 0.029666199887420187 | 0.061955941261482975 | 0 | 0 | 44.40044742729307 | 156.189537724504 | MKI67 |
| tolazoline PC3 DOWN | 1/153 | 0.030252709124753842 | 0.06275393041418532 | 0 | 0 | 43.51754385964912 | 152.23174641469322 | ENO1 |
| N-NITROSODIETHYLAMINE CTD 00005817 | 1/157 | 0.031034307833845202 | 0.06391665024567442 | 0 | 0 | 42.39316239316239 | 147.21712334479366 | MKI67 |
| Pyruvic acid BOSS | 1/158 | 0.031229633670524955 | 0.06391665024567442 | 0 | 0 | 42.12101910828026 | 146.00778835295287 | ENO1 |
| epivincamine HL60 DOWN | 1/160 | 0.0316201967624613 | 0.06428741990778555 | 0 | 0 | 41.58700209643606 | 143.6398095102211 | ENO1 |
| liothyronine CTD 00006943 | 1/162 | 0.032010641763302816 | 0.06465307250877608 | 0 | 0 | 41.06625258799172 | 141.3371825935152 | ENO1 |
| Retinoic acid CTD 00006918 | 3/4258 | 0.03242173556542055 | 0.06505537789924254 | 0 | 0 | 11.098237367802586 | 38.055037220765556 | FABP5;ENO1;MKI67 |
| DL-METHIONINE BOSS | 1/167 | 0.03298623774343714 | 0.06523019664500183 | 0 | 0 | 39.81927710843374 | 135.8500277677097 | ENO1 |
| GLYCOGEN BOSS | 1/167 | 0.03298623774343714 | 0.06523019664500183 | 0 | 0 | 39.81927710843374 | 135.8500277677097 | ENO1 |
| diphenylpyraline BOSS | 1/168 | 0.03318126841962593 | 0.06523019664500183 | 0 | 0 | 39.578842315369265 | 134.79642455118795 | ENO1 |
| captopril HL60 DOWN | 1/170 | 0.03357124127006609 | 0.06523019664500183 | 0 | 0 | 39.10650887573964 | 132.7308344608708 | ENO1 |
| bicalutamide CTD 00002279 | 1/170 | 0.03357124127006609 | 0.06523019664500183 | 0 | 0 | 39.10650887573964 | 132.7308344608708 | MKI67 |
| 14-Deoxy-11,12-didehydroandrographolide CTD 00004394 | 1/172 | 0.033961096146849654 | 0.06557268249737637 | 0 | 0 | 38.64522417153996 | 130.71900267520095 | ENO1 |
| resveratrol CTD 00002483 | 2/1601 | 0.0344518522209748 | 0.06581689405247448 | 0 | 0 | 11.505315822388994 | 38.75211866424134 | ENO1;MKI67 |
| ambroxol PC3 UP | 1/175 | 0.03454565731094125 | 0.06581689405247448 | 0 | 0 | 37.97318007662835 | 127.79772858341224 | ENO1 |
| dimethyl sulfoxide BOSS | 1/177 | 0.034935217346908146 | 0.06581689405247448 | 0 | 0 | 37.53787878787879 | 125.91180025535921 | ENO1 |
| clindamycin HL60 DOWN | 2/1626 | 0.035474471432271816 | 0.06581689405247448 | 0 | 0 | 11.312807881773399 | 37.77280887403658 | ENO1;MKI67 |
| folic acid BOSS | 1/181 | 0.03571398376740333 | 0.06581689405247448 | 0 | 0 | 36.696296296296296 | 122.27987427585897 | ENO1 |
| hemin BOSS | 1/181 | 0.03571398376740333 | 0.06581689405247448 | 0 | 0 | 36.696296296296296 | 122.27987427585897 | ENO1 |
| ZINC CTD 00007011 | 2/1642 | 0.03613578975840923 | 0.06581689405247448 | 0 | 0 | 11.192682926829269 | 37.16498465265242 | FABP5;ENO1 |
| sucrose BOSS | 1/185 | 0.036492278796295255 | 0.06581689405247448 | 0 | 0 | 35.891304347826086 | 118.82371114280049 | ENO1 |
| Ethylparaben BOSS | 1/186 | 0.036686778924747046 | 0.06581689405247448 | 0 | 0 | 35.69549549549549 | 117.98570753539191 | ENO1 |
| lactic acid BOSS | 1/186 | 0.036686778924747046 | 0.06581689405247448 | 0 | 0 | 35.69549549549549 | 117.98570753539191 | ENO1 |
| Coke BOSS | 1/189 | 0.03727010263208895 | 0.06581689405247448 | 0 | 0 | 35.12056737588652 | 115.53134748782352 | ENO1 |
| ciglitazone CTD 00001835 | 1/190 | 0.03746448498573523 | 0.06581689405247448 | 0 | 0 | 34.932980599647266 | 114.73254920062688 | MKI67 |
| HEXACHLOROETHANE BOSS | 1/190 | 0.03746448498573523 | 0.06581689405247448 | 0 | 0 | 34.932980599647266 | 114.73254920062688 | ENO1 |
| Isoguanine BOSS | 1/192 | 0.03785316139800214 | 0.06581689405247448 | 0 | 0 | 34.56369982547993 | 113.16296267411514 | ENO1 |
| ipratropium bromide HL60 DOWN | 1/192 | 0.03785316139800214 | 0.06581689405247448 | 0 | 0 | 34.56369982547993 | 113.16296267411514 | ENO1 |
| diazepam BOSS | 1/193 | 0.03804745545598449 | 0.06581689405247448 | 0 | 0 | 34.38194444444444 | 112.39186264276356 | ENO1 |
| ethisterone BOSS | 1/193 | 0.03804745545598449 | 0.06581689405247448 | 0 | 0 | 34.38194444444444 | 112.39186264276356 | ENO1 |
| COPPER CTD 00005706 | 2/1688 | 0.0380666084245778 | 0.06581689405247448 | 0 | 0 | 10.860023724792407 | 35.49509485522362 | ENO1;MKI67 |
| PLATINUM BOSS | 1/194 | 0.03824172009366363 | 0.06581689405247448 | 0 | 0 | 34.2020725388601 | 111.62968920243314 | ENO1 |
| picrotoxinin PC3 DOWN | 1/196 | 0.03863016109224715 | 0.06581689405247448 | 0 | 0 | 33.847863247863245 | 110.1315350069817 | ENO1 |
| LAMININ BOSS | 1/197 | 0.038824337461276164 | 0.06581689405247448 | 0 | 0 | 33.673469387755105 | 109.39526854046228 | ENO1 |
| Acid red 87 BOSS | 1/198 | 0.03901848442198813 | 0.06581689405247448 | 0 | 0 | 33.500846023688666 | 108.66735708130022 | ENO1 |
| hydrocortisone BOSS | 1/198 | 0.03901848442198813 | 0.06581689405247448 | 0 | 0 | 33.500846023688666 | 108.66735708130022 | ENO1 |
| L-thyroxine BOSS | 1/201 | 0.03960074884002518 | 0.06643404313599853 | 0 | 0 | 32.99333333333333 | 106.53241322802339 | ENO1 |
| monobenzone PC3 DOWN | 1/204 | 0.04018274863527793 | 0.06656753485904819 | 0 | 0 | 32.500821018062396 | 104.46795821414041 | MKI67 |
| norgestrel CTD 00006422 | 1/205 | 0.04037668977225679 | 0.06656753485904819 | 0 | 0 | 32.33986928104575 | 103.79489604079704 | MKI67 |
| Capsaicin CTD 00005570 | 1/206 | 0.04057060152551671 | 0.06656753485904819 | 0 | 0 | 32.18048780487805 | 103.1291817580333 | FABP5 |
| epinephrine BOSS | 1/207 | 0.0407644838876256 | 0.06656753485904819 | 0 | 0 | 32.02265372168285 | 102.47070087023805 | ENO1 |
| 8-azaguanine PC3 DOWN | 1/207 | 0.0407644838876256 | 0.06656753485904819 | 0 | 0 | 32.02265372168285 | 102.47070087023805 | MKI67 |
| Alitretinoin CTD 00003402 | 1/212 | 0.0417334549875237 | 0.0677454655347846 | 0 | 0 | 31.255924170616115 | 99.2829489057505 | FABP5 |
| LUCANTHONE CTD 00006227 | 1/213 | 0.04192716108015985 | 0.0677454655347846 | 0 | 0 | 31.10691823899371 | 98.66558977550699 | MKI67 |
| Decitabine CTD 00000750 | 2/1800 | 0.04294825972486841 | 0.06889716554700573 | 0 | 0 | 10.121245828698553 | 31.859244166706944 | FABP5;ENO1 |
| dexibuprofen PC3 DOWN | 1/219 | 0.04308878105871368 | 0.06889716554700573 | 0 | 0 | 30.24159021406728 | 95.0944571247856 | ENO1 |
| COUMESTROL CTD 00005717 | 2/1812 | 0.043486253687520335 | 0.06917243462211785 | 0 | 0 | 10.047513812154696 | 31.5020745294151 | FABP5;MKI67 |
| ethanol CTD 00005337 | 1/231 | 0.04540885191709206 | 0.0718583378275632 | 0 | 0 | 28.6463768115942 | 88.5759783378648 | ENO1 |
| dorzolamide HL60 DOWN | 1/238 | 0.046760276892818704 | 0.0736174615697197 | 0 | 0 | 27.790436005625878 | 85.11435809030968 | ENO1 |
| tolnaftate PC3 DOWN | 1/240 | 0.04714613463404288 | 0.07384624149311819 | 0 | 0 | 27.555090655509066 | 84.16711405645033 | ENO1 |
| digoxigenin HL60 UP | 1/244 | 0.04791749867966518 | 0.07467346240942746 | 0 | 0 | 27.0960219478738 | 82.32515319610377 | MKI67 |
| rapamycin CTD 00007350 | 1/247 | 0.04849571431961073 | 0.07519284998040654 | 0 | 0 | 26.761517615176153 | 80.98784150050393 | MKI67 |
| HEMATOXYLIN BOSS | 1/251 | 0.04926625878446066 | 0.07600372586346443 | 0 | 0 | 26.328 | 79.26086098920652 | ENO1 |
| PNU-0293363 MCF7 UP | 1/255 | 0.050036335166732955 | 0.07676280451008627 | 0 | 0 | 25.908136482939632 | 77.59501991810856 | ENO1 |
| calcitriol CTD 00005558 | 2/1958 | 0.05025838340888384 | 0.07676280451008627 | 0 | 0 | 9.222903885480573 | 27.581812643961186 | MKI67;MPHOSPH6 |
| zuclopenthixol PC3 DOWN | 1/260 | 0.05099827270969593 | 0.0770367929613035 | 0 | 0 | 25.401544401544403 | 75.5940693704739 | ENO1 |
| cyclopenthiazide PC3 DOWN | 1/261 | 0.05119057252151764 | 0.0770367929613035 | 0 | 0 | 25.3025641025641 | 75.20427835191589 | ENO1 |
| ascorbic acid CTD 00005445 | 1/261 | 0.05119057252151764 | 0.0770367929613035 | 0 | 0 | 25.3025641025641 | 75.20427835191589 | MKI67 |
| digoxin HL60 UP | 1/267 | 0.05234375776008811 | 0.07800744481721869 | 0 | 0 | 24.724310776942357 | 72.93480286504084 | MKI67 |
| niclosamide HL60 DOWN | 1/267 | 0.05234375776008811 | 0.07800744481721869 | 0 | 0 | 24.724310776942357 | 72.93480286504084 | MKI67 |
| 2-Nonenal, 4-hydroxy-, (2E,4R)- CTD 00001295 | 1/274 | 0.053687811646846155 | 0.07962395253904236 | 0 | 0 | 24.08180708180708 | 70.42891306405565 | ENO1 |
| melphalan CTD 00006262 | 1/279 | 0.054646974475143 | 0.08055256884416773 | 0 | 0 | 23.642685851318944 | 68.72601154931702 | FABP5 |
| PHA-00665752 MCF7 DOWN | 1/280 | 0.05483871950629008 | 0.08055256884416773 | 0 | 0 | 23.55675029868578 | 68.39369767110742 | ENO1 |
| arbutin CTD 00005438 | 1/282 | 0.055222122083059724 | 0.08072948323571112 | 0 | 0 | 23.386714116251483 | 67.73708333777427 | MKI67 |
| helveticoside HL60 UP | 1/301 | 0.05885863177620021 | 0.08551070227528416 | 0 | 0 | 21.884444444444444 | 61.99024459456627 | MKI67 |
| lycorine PC3 DOWN | 1/302 | 0.059049735773160396 | 0.08551070227528416 | 0 | 0 | 21.81063122923588 | 61.71045933577671 | MKI67 |
| pergolide HL60 UP | 1/312 | 0.060959175517745774 | 0.08786134687299507 | 0 | 0 | 21.09860664523044 | 59.02442585438528 | MKI67 |
| proscillaridin HL60 UP | 1/323 | 0.06305620122899361 | 0.09045912980047215 | 0 | 0 | 20.366459627329192 | 56.287372395940324 | MKI67 |
| mifepristone CTD 00007083 | 1/334 | 0.06514971281846585 | 0.09286729808821292 | 0 | 0 | 19.68268268268268 | 53.75473269300094 | MKI67 |
| amikacin PC3 DOWN | 1/335 | 0.06533985793828662 | 0.09286729808821292 | 0 | 0 | 19.622754491017965 | 53.533877455276205 | ENO1 |
| strophanthidin HL60 UP | 1/344 | 0.06704985903942734 | 0.09485855633688568 | 0 | 0 | 19.099125364431487 | 51.61192499300385 | MKI67 |
| mebendazole HL60 UP | 1/346 | 0.06742954041992155 | 0.0949581142610822 | 0 | 0 | 18.98647342995169 | 51.200292642815555 | MKI67 |
| 5-azacytidine CTD 00005455 | 1/352 | 0.06856788928476143 | 0.09612028315261077 | 0 | 0 | 18.65622032288699 | 49.997382067970975 | MKI67 |
| MG-262 PC3 DOWN | 1/360 | 0.0700840662985625 | 0.09779912888026676 | 0 | 0 | 18.2330547818013 | 48.46455013579165 | MKI67 |
| diltiazem PC3 DOWN | 1/364 | 0.0708414603136072 | 0.09840872541301995 | 0 | 0 | 18.028466483011936 | 47.726954965901264 | ENO1 |
| scopolamine PC3 DOWN | 1/368 | 0.07159839157932052 | 0.09856818930426636 | 0 | 0 | 17.8283378746594 | 47.007669495537755 | ENO1 |
| 0175029-0000 MCF7 UP | 1/368 | 0.07159839157932052 | 0.09856818930426636 | 0 | 0 | 17.8283378746594 | 47.007669495537755 | ENO1 |
| nocodazole HL60 UP | 1/373 | 0.07254390522266853 | 0.09942401296142517 | 0 | 0 | 17.584229390681003 | 46.13333908403417 | MKI67 |
| sanguinarine MCF7 DOWN | 1/376 | 0.07311086663642512 | 0.09975571581058894 | 0 | 0 | 17.44088888888889 | 45.621498145459356 | MKI67 |
| ouabain HL60 UP | 1/379 | 0.07367756808507164 | 0.10008413009786281 | 0 | 0 | 17.299823633156965 | 45.11892429188263 | MKI67 |
| daunorubicin PC3 DOWN | 1/381 | 0.07405522465425209 | 0.10015398224165371 | 0 | 0 | 17.20701754385965 | 44.78890625688341 | ENO1 |
| Methaneseleninic acid CTD 00000412 | 1/402 | 0.07801365068255263 | 0.10458598584953561 | 0 | 0 | 16.288445552784705 | 41.54973086969775 | MKI67 |
| Dronabinol CTD 00006853 | 1/402 | 0.07801365068255263 | 0.10458598584953561 | 0 | 0 | 16.288445552784705 | 41.54973086969775 | ENO1 |
| MG-262 MCF7 DOWN | 1/407 | 0.07895425941288749 | 0.10538677234676722 | 0 | 0 | 16.08374384236453 | 40.83480154182249 | MKI67 |
| digitoxigenin HL60 UP | 1/421 | 0.08158413529015146 | 0.10795831695722628 | 0 | 0 | 15.536507936507936 | 38.93636036036191 | MKI67 |
| dexamethasone CTD 00005779 | 1/421 | 0.08158413529015146 | 0.10795831695722628 | 0 | 0 | 15.536507936507936 | 38.93636036036191 | MKI67 |
| Vorinostat CTD 00003560 | 1/425 | 0.08233449290606551 | 0.10802003983829962 | 0 | 0 | 15.38679245283019 | 38.420284482564895 | ENO1 |
| Zoledronic acid CTD 00003127 | 1/425 | 0.08233449290606551 | 0.10802003983829962 | 0 | 0 | 15.38679245283019 | 38.420284482564895 | MKI67 |
| lanatoside C HL60 UP | 1/432 | 0.08364651226451049 | 0.10927437985193499 | 0 | 0 | 15.131477184841454 | 37.543548548533906 | MKI67 |
| deptropine HL60 DOWN | 1/435 | 0.08420837540090606 | 0.10954225105117865 | 0 | 0 | 15.02457757296467 | 37.17772962854749 | ENO1 |
| theophylline CTD 00006862 | 1/472 | 0.09111679767747995 | 0.1180289320126006 | 0 | 0 | 13.818117480537863 | 33.102863315543075 | MPHOSPH6 |
| azacitidine MCF7 DOWN | 1/482 | 0.09297721059339711 | 0.11993278845450805 | 0 | 0 | 13.523908523908524 | 32.12470398208089 | MKI67 |
| paclitaxel CTD 00007144 | 1/493 | 0.09502036448564323 | 0.12205544726816935 | 0 | 0 | 13.214092140921409 | 31.10153359042112 | MPHOSPH6 |
| ajmaline HL60 DOWN | 1/496 | 0.09557698870606544 | 0.12225889805317537 | 0 | 0 | 13.131986531986533 | 30.831582534335432 | FABP5 |
| Dasatinib CTD 00004330 | 1/502 | 0.09668946679188478 | 0.12316873985522252 | 0 | 0 | 12.970725216234198 | 30.302867281258724 | MKI67 |
| oxygen CTD 00006454 | 1/524 | 0.10075977480730314 | 0.1278233506852978 | 0 | 0 | 12.411089866156788 | 28.48365059523648 | ENO1 |
| chlorhexidine PC3 DOWN | 1/534 | 0.10260536056653674 | 0.12962899462521307 | 0 | 0 | 12.17198248905566 | 27.713962131683417 | ENO1 |
| fisetin PC3 DOWN | 1/541 | 0.10389557923179935 | 0.13072107714820655 | 0 | 0 | 12.009876543209877 | 27.194791298547404 | MKI67 |
| piroxicam CTD 00006571 | 1/549 | 0.10536841075345059 | 0.13203306980126256 | 0 | 0 | 11.829683698296837 | 26.62024727357418 | MKI67 |
| anisomycin PC3 DOWN | 1/558 | 0.10702317526077482 | 0.13352246349672411 | 0 | 0 | 11.63315380011969 | 25.996723694885414 | MKI67 |
| atrazine CTD 00005450 | 2/2968 | 0.10742686802505164 | 0.13352246349672411 | 0 | 0 | 5.74173971679029 | 12.809505284889985 | MKI67;MPHOSPH6 |
| Irinotecan hydrochloride CTD 00002224 | 1/565 | 0.10830862645707154 | 0.13407559807387484 | 0 | 0 | 11.484633569739954 | 25.527704412762255 | ENO1 |
| vorinostat HL60 DOWN | 1/569 | 0.10904254658434588 | 0.13444201526664332 | 0 | 0 | 11.401408450704226 | 25.265716515865964 | MKI67 |
| sulfaguanidine PC3 DOWN | 1/583 | 0.1116076998661258 | 0.13705425543560248 | 0 | 0 | 11.119129438717067 | 24.381640490013528 | ENO1 |
| 4-Hydroxytamoxifen CTD 00000850 | 1/586 | 0.11215665415822744 | 0.13708096058603522 | 0 | 0 | 11.06039886039886 | 24.198589729651637 | ENO1 |
| trichostatin A MCF7 DOWN | 1/588 | 0.11252248230514945 | 0.13708096058603522 | 0 | 0 | 11.021578648495174 | 24.077765344224307 | ENO1 |
| azacitidine PC3 DOWN | 1/595 | 0.113801990631087 | 0.13809174357210952 | 0 | 0 | 10.88776655443322 | 23.66233150002798 | MKI67 |
| AFLATOXIN B1 CTD 00007128 | 2/3081 | 0.11481319921212406 | 0.13877028408709483 | 0 | 0 | 5.494316336472881 | 11.892166544099183 | FABP5;MKI67 |
| benzene CTD 00005481 | 1/615 | 0.11745010590355782 | 0.14117027700374038 | 0 | 0 | 10.522258414766558 | 22.53595926929282 | MKI67 |
| sulpiride PC3 DOWN | 1/617 | 0.11781429691661129 | 0.14117027700374038 | 0 | 0 | 10.487012987012987 | 22.42800469687245 | ENO1 |
| methotrexate CTD 00006299 | 1/619 | 0.11817837521160025 | 0.14117027700374038 | 0 | 0 | 10.451995685005393 | 22.320865384865435 | MPHOSPH6 |
| quercetin CTD 00006679 | 2/3158 | 0.11994664349137434 | 0.1427272075653175 | 0 | 0 | 5.335868187579214 | 11.315819807182907 | ENO1;MKI67 |
| neostigmine bromide PC3 DOWN | 1/650 | 0.12380719139900447 | 0.14640158884139412 | 0 | 0 | 9.936825885978429 | 20.758325706963554 | ENO1 |
| troglitazone CTD 00002415 | 1/651 | 0.12398831628261392 | 0.14640158884139412 | 0 | 0 | 9.921025641025642 | 20.710815073454185 | MKI67 |
| scriptaid MCF7 DOWN | 1/670 | 0.12742435666247415 | 0.1498822892543278 | 0 | 0 | 9.629795714997508 | 19.83961686319277 | MKI67 |
| raloxifene CTD 00007367 | 1/686 | 0.13031001583705082 | 0.1526915071067733 | 0 | 0 | 9.397080291970802 | 19.14973605005474 | MKI67 |
| 0175029-0000 PC3 DOWN | 2/3326 | 0.13141773914860527 | 0.15320946461042254 | 0 | 0 | 5.015643802647412 | 10.178618033746556 | ENO1;MKI67 |
| digitoxigenin PC3 DOWN | 1/694 | 0.13175015849235033 | 0.15320946461042254 | 0 | 0 | 9.284752284752285 | 18.818780564370044 | ENO1 |
| Fonofos CTD 00005884 | 1/712 | 0.13498393957217353 | 0.15582494846315836 | 0 | 0 | 9.041256446319737 | 18.1060154014639 | FABP5 |
| meclofenoxate HL60 DOWN | 1/713 | 0.13516332880215814 | 0.15582494846315836 | 0 | 0 | 9.02808988764045 | 18.0676579879656 | MKI67 |
| TERBUFOS CTD 00000658 | 1/715 | 0.1355220235819651 | 0.15582494846315836 | 0 | 0 | 9.00186741363212 | 17.991322298204356 | FABP5 |
| doxorubicin CTD 00005874 | 1/750 | 0.14178113941000473 | 0.16241346939877405 | 0 | 0 | 8.565643079661772 | 16.732732629645426 | MKI67 |
| parathion CTD 00006472 | 1/776 | 0.146408719152892 | 0.16709099174698083 | 0 | 0 | 8.267096774193549 | 15.884012198403969 | FABP5 |
| Vitinoin CTD 00007069 | 1/780 | 0.14711898998081238 | 0.16727974045966446 | 0 | 0 | 8.22293538724861 | 15.759367205427294 | FABP5 |
| emetine PC3 UP | 1/801 | 0.1508406423747059 | 0.17044996611585925 | 0 | 0 | 7.998333333333333 | 15.12909823136499 | ENO1 |
| tamoxifen CTD 00006827 | 1/802 | 0.15101755955541926 | 0.17044996611585925 | 0 | 0 | 7.987931751976696 | 15.10005996065182 | MKI67 |
| Caspan CTD 00000180 | 1/808 | 0.15207848222909875 | 0.17101865950305245 | 0 | 0 | 7.92606361007848 | 14.927619754548486 | MKI67 |
| ARSENIC CTD 00005442 | 1/853 | 0.16000374126434233 | 0.17927426484727407 | 0 | 0 | 7.489827856025039 | 13.725544563752972 | ENO1 |
| lycorine HL60 UP | 1/862 | 0.16158210233360765 | 0.18038438333242746 | 0 | 0 | 7.408052651955091 | 13.502967906228978 | MPHOSPH6 |
| Bortezomib CTD 00003736 | 1/878 | 0.1643825842134824 | 0.1828458454838373 | 0 | 0 | 7.266818700114025 | 13.120667997185004 | MKI67 |
| 8-azaguanine HL60 DOWN | 1/897 | 0.16769903772862843 | 0.1858613883851586 | 0 | 0 | 7.105654761904762 | 12.68774592672443 | MKI67 |
| 0179445-0000 MCF7 DOWN | 1/911 | 0.1701364159870504 | 0.18788445938138298 | 0 | 0 | 6.991208791208791 | 12.38251242624733 | MKI67 |
| Enterolactone CTD 00001393 | 1/971 | 0.180521740973597 | 0.19863861820392215 | 0 | 0 | 6.538144329896907 | 11.192675822270862 | MKI67 |
| irinotecan PC3 DOWN | 1/999 | 0.18533472548597407 | 0.203206288300693 | 0 | 0 | 6.345357381429526 | 10.695682127896339 | MKI67 |
| diazinon CTD 00005790 | 1/1027 | 0.1901264780114699 | 0.2077182517776557 | 0 | 0 | 6.163092917478882 | 10.231139494352021 | ENO1 |
| anisomycin HL60 DOWN | 1/1034 | 0.19132110598091992 | 0.20828219693667524 | 0 | 0 | 6.119070667957406 | 10.11973179463795 | MKI67 |
| CADMIUM CTD 00005555 | 1/1142 | 0.20958554509725114 | 0.2265590223410426 | 0 | 0 | 5.508326029798423 | 8.60743856039478 | ENO1 |
| anisomycin HL60 UP | 1/1142 | 0.20958554509725114 | 0.2265590223410426 | 0 | 0 | 5.508326029798423 | 8.60743856039478 | MPHOSPH6 |
| benzo[a]pyrene CTD 00005488 | 2/4424 | 0.21415935176258166 | 0.23069095084600902 | 0 | 0 | 3.521935775667119 | 5.427425969311865 | FABP5;ENO1 |
| 5-Fluorouracil CTD 00005987 | 1/1202 | 0.21959778250819134 | 0.23572209520984172 | 0 | 0 | 5.216486261448793 | 7.907972344229623 | MKI67 |
| vincristine CTD 00006988 | 1/1245 | 0.22671448631120295 | 0.24202292416735255 | 0 | 0 | 5.02465166130761 | 7.456903752270924 | MKI67 |
| testosterone CTD 00006844 | 1/1247 | 0.22704430671074113 | 0.24202292416735255 | 0 | 0 | 5.016051364365971 | 7.436848398870614 | MKI67 |
| irinotecan MCF7 DOWN | 1/1272 | 0.23115816631274663 | 0.24555556075437102 | 0 | 0 | 4.91083136637818 | 7.19266438488164 | MKI67 |
| arsenite CTD 00000779 | 1/1300 | 0.23574616991968034 | 0.24956577298393745 | 0 | 0 | 4.797793174236593 | 6.932809236675803 | ENO1 |
| GW-8510 PC3 DOWN | 1/1312 | 0.2377061582905896 | 0.2507759126983196 | 0 | 0 | 4.750826341215357 | 6.825607207288126 | MKI67 |
| glibenclamide HL60 DOWN | 1/1333 | 0.2411270636744635 | 0.2535137279043161 | 0 | 0 | 4.67067067067067 | 6.643707916998492 | MKI67 |
| cyclosporin A CTD 00007121 | 2/4825 | 0.24703061115535938 | 0.2580536986221116 | 0 | 0 | 3.145967240306863 | 4.398826729339362 | ENO1;MKI67 |
| Disodium selenite CTD 00007229 | 1/1370 | 0.24712634330586583 | 0.2580536986221116 | 0 | 0 | 4.535427319211103 | 6.339872303170645 | MKI67 |
| alsterpaullone MCF7 DOWN | 1/1469 | 0.2630035949309563 | 0.2737020462501817 | 0 | 0 | 4.20708446866485 | 5.618929755772148 | MKI67 |
| lobeline HL60 DOWN | 1/1510 | 0.2695049413337103 | 0.2781326815789538 | 0 | 0 | 4.08371990280539 | 5.354445118314422 | MKI67 |
| VITAMIN E CTD 00006994 | 1/1510 | 0.2695049413337103 | 0.2781326815789538 | 0 | 0 | 4.08371990280539 | 5.354445118314422 | ENO1 |
| camptothecin MCF7 DOWN | 1/1513 | 0.26997895475742095 | 0.2781326815789538 | 0 | 0 | 4.074955908289241 | 5.335793184402607 | MKI67 |
| latamoxef HL60 DOWN | 1/1578 | 0.28019271607508184 | 0.2876895111540138 | 0 | 0 | 3.8932572394842526 | 4.953304135937465 | MKI67 |
| POTASSIUM CHROMATE CTD 00001284 | 1/1897 | 0.3287738389369496 | 0.33644522851214514 | 0 | 0 | 3.1821378340365682 | 3.5397629807998428 | MPHOSPH6 |
| progesterone CTD 00006624 | 1/1915 | 0.3314397056158128 | 0.33804647715632735 | 0 | 0 | 3.1490769766631836 | 3.4775552103253307 | MKI67 |
| Copper sulfate CTD 00007279 | 2/6016 | 0.34970660616035415 | 0.3554964506332077 | 0 | 0 | 2.32490854672431 | 2.4426901437940054 | FABP5;MKI67 |
| (-)-Epigallocatechin gallate CTD 00002033 | 1/2114 | 0.36038615672258445 | 0.36514372974862513 | 0 | 0 | 2.821107430194037 | 2.8791634650745204 | MPHOSPH6 |
| ETHYL METHANESULFONATE CTD 00005938 | 1/2315 | 0.38865892632273824 | 0.39249437625355477 | 0 | 0 | 2.547104580812446 | 2.407149121026783 | FABP5 |
| VALPROIC ACID CTD 00006977 | 2/8312 | 0.5515795078074169 | 0.5551964226127114 | 0 | 0 | 1.4062575210589652 | 0.8366800307207815 | FABP5;ENO1 |
| Tetradioxin CTD 00006848 | 1/3768 | 0.5661510994984884 | 0.5680012664903136 | 0 | 0 | 1.4360676046367578 | 0.8169706404592676 | ENO1 |
| estradiol CTD 00005920 | 1/4336 | 0.6237683149811166 | 0.6237683149811166 | 0 | 0 | 1.2042291426374472 | 0.5683675785486184 | MKI67 |
